# Ceramic Mineral Waste-Forms for Nuclear Waste Immobilization

**DOI:** 10.3390/ma12162638

**Published:** 2019-08-19

**Authors:** Albina I. Orlova, Michael I. Ojovan

**Affiliations:** 1 Lobachevsky State University of Nizhny Novgorod, 23 Gagarina av., 603950 Nizhny Novgorod, Russian Federation; 2 Department of Radiochemistry, Lomonosov Moscow State University, Moscow 119991, Russia; 3 Imperial College London, South Kensington Campus, Exhibition Road, London SW7 2AZ, UK

**Keywords:** crystalline ceramics, nuclear waste, immobilization, sintering, spark plasma sintering

## Abstract

Crystalline ceramics are intensively investigated as effective materials in various nuclear energy applications, such as inert matrix and accident tolerant fuels and nuclear waste immobilization. This paper presents an analysis of the current status of work in this field of material sciences. We have considered inorganic materials characterized by different structures, including simple oxides with fluorite structure, complex oxides (pyrochlore, murataite, zirconolite, perovskite, hollandite, garnet, crichtonite, freudenbergite, and P-pollucite), simple silicates (zircon/thorite/coffinite, titanite (sphen), britholite), framework silicates (zeolite, pollucite, nepheline /leucite, sodalite, cancrinite, micas structures), phosphates (monazite, xenotime, apatite, kosnarite (NZP), langbeinite, thorium phosphate diphosphate, struvite, meta-ankoleite), and aluminates with a magnetoplumbite structure. These materials can contain in their composition various cations in different combinations and ratios: Li–Cs, Tl, Ag, Be–Ba, Pb, Mn, Co, Ni, Cu, Cd, B, Al, Fe, Ga, Sc, Cr, V, Sb, Nb, Ta, La, Ce, rare-earth elements (REEs), Si, Ti, Zr, Hf, Sn, Bi, Nb, Th, U, Np, Pu, Am and Cm. They can be prepared in the form of powders, including nano-powders, as well as in form of monolith (bulk) ceramics. To produce ceramics, cold pressing and sintering (frittage), hot pressing, hot isostatic pressing and spark plasma sintering (SPS) can be used. The SPS method is now considered as one of most promising in applications with actual radioactive substances, enabling a densification of up to 98–99.9% to be achieved in a few minutes. Characteristics of the structures obtained (e.g., syngony, unit cell parameters, drawings) are described based upon an analysis of 462 publications.

## 1. Introduction

Crystalline ceramics, aiming to immobilize high-level radioactive waste (HLW), are important for the current stage of development of modern nuclear technology in the world.

The crystal-chemical principle is used to design multicomponent ceramics with needed structures. The approach to designing mineral-like crystalline materials is based upon the structural features of materials and isomorphism concept. The choice of the structural forms of compounds for discussion here was based upon the following criteria:

(1) The ability of the structure to include various cations in different combinations and ratios.
(2) Known high stability of structure to the action of the destructive factors of the environment during their prolonged exposure (“mineral-like” compounds preferred while “the nature suggests”) such as high temperatures, thermal “stresses”, radiation levels, the corrosive action of water and other chemical solutions. Criteria for the resistance of materials to such effects are justified by the features of the crystal structure of materials including small interatomic distances, and the possibility of their controlled variation in the desired direction when cations and/or anions of given sizes are included in the crystallographic positions. Most of the crystalline matrices discussed in the present work meet these criteria in full or in part. Herewith the classification criteria for crystalline ceramics were based on considering first simple and then more complex structures, e.g., starting with oxides (from simple oxides to complex oxides) and moving to salt compositions (from simple salt to complex ones).

The concept of immobilizing the radioactive elements of nuclear waste in an assemblage of mineral phases was originally introduced by Hatch [1] at Brookhaven National Laboratory in 1953. The feasibility of making a ceramic of natural, mineralogically-stable phases was demonstrated by McCarthy [2,3] and Roy [4] at the Pennsylvania State University between 1973 and 1976. Since that time, a number of other mineralogic-ceramic assemblages have been developed [5]. Among these are the Sandia titanate-based ceramic [6], the Australian ceramic “SYNROC” [7,8,9,10], the silicate-phosphate supercalcine ceramics [11], the alumina-based tailored ceramics [12,13] and the Pu pyrochlores [14,15]. The structural types of monazite, kosnarite (NZP), langbeinite and other ones were considered as matrices for the incorporation of simulated wastes containing f-elements and that also contain uni-, bi-, and trivalent elements involved in radiochemical processes [16,17,18,19,20,21,22,23,24,25,26,27]. Cold pressing and sintering, as well as hot isostatic pressing often result in ceramics containing an intergranular glassy phase with radionuclides preferentially migrating to the glassy phase [28,29,30,31,32,33,34,35,36]. The radionuclides that are incorporated in the intergranular glassy phase(s) will then have leaching rates at about the same order as those from a glassy waste-form.

Crystalline waste-forms synthesized at moderate temperatures such as within 700 to 750 °C have not been investigated as intensely as those formed at high temperatures [11], although crystalline waste-forms made from clay have been studied almost continuously since the 1953 work of Hatch [1,11]. Supercalcine ceramics synthesized at high temperatures often contained sodalite-cancrinite mineral assemblages. Roy [37] proposed in 1981 a low solubility phase assemblage as a waste-form [37] using a low temperature hydrothermal process. The assemblage consisted of micas, apatite, pollucite, sodalite-cancrinite and nepheline, many of which could be produced using various clay minerals such as kaolin, bentonite and illite mixed with radioactive waste. However there were no continuous commercial technologies available at that time that could process the waste/clay mixtures in a hydrothermal environment, and clay-based crystalline waste-forms were not pursued. The situation changed in 1999 when Studsvik had built in Erwin a commercial facility to continuously process radioactive wastes by pyrolysis at moderate temperatures in a hydrothermal steam environment [38,39]. This facility utilizes Fluidized Bed Steam Reforming (FBSR) technology to pyrolyze ^137^Cs- and ^60^Co-contaning spent organic ion exchange resins produced by commercial nuclear facilities. FBSR technology can also process a wide variety of solid and liquid radioactive wastes, including spent organic ion exchange resins, charcoal, graphite, sludge, oils, solvents and cleaning solutions with contaminations up to radiation levels reaching 4 Sv/h (400 R/hr). The waste organics are destroyed, creating steam and CO_2_. The clay serves in the FBSR process as a mineralizing agent, and feldspathoid minerals (sodalite, nosean and nepheline) are formed by the nanoscale reaction of waste components with clay. The phases formed act as hosts for waste contaminants such as Cl, I, F, ^99^Tc from SO_4_ alkali (Na, K, Cs) bearing wastes [40,41,42,43,44]. The mineralization occurs at moderate temperatures used within the range when most clays become amorphous at the nanoscale level, e.g., kaolin, bentonite (montmorillonite), and illite. The octahedral Al^3+^ cation in the clay structure is destabilized, and clays become amorphous as confirmed by X-ray diffraction (XRD) analysis, losing their hydroxyl (OH–) groups. The alkalis from waste act as activators of unstable Al^3+^ cations, and form new mineral phases catalyzing the mineralization. In the absence of steam many of these mineral phases can only be formed if temperatures are above 1200 °C.

Many of the compounds under consideration have structures similar to those of natural minerals (the so-called mineral-like compounds). Others of the discussed ones are not structural analogs of any known minerals (that its, of what is known today, as there are examples of compounds being developed for the radioactive waste immobilization that were obtained synthetically, and many years later a mineral was discovered, whose structural analog they became. For example, the mineral kosnarite KrZr_2_(PO_4_)_3_ was discovered in 1991, and then kosnarite-like compounds (for example, NZP and NASICON) were synthesized and investigated many years before the discovery of this mineral).

Ceramic waste-forms can range from single phase, i.e., UO_2_ and single phase solid solutions, i.e., (U, Th, Pu)O_2_, to multiphase ceramics formulated in a such way that each waste radionuclide can substitute on a given host lattice in the various phases used.

## 2. Theoretical Aspects of Substitution

The crystal-chemical substitutions in crystalline waste-forms must be electrically balanced [45,46] which is important when relying on the long range order (LRO) of crystals accounting for the size and coordination of the crystallographic site, which will act as host to a given radionuclide, or its decay product upon transmutation (see [15] for natural analogs). Moreover, if a monovalent cation transmutes to a divalent one, the substitutions must be coupled to retain the electrical balance of the host phase without destroying the integrity of the phase. It means that the lattice site must be of suitable size and have a bond coordination able to accept the cation resulting from transmutation. The bond system of a crystalline ceramic can only maintain its charge balance if:

(1) Sufficient lattice vacancies exist in the structure or,
(2) A variable valence cation such as Fe or Ti is present in a neighboring lattice site balancing the charge.

Both above ways assume that the variable valence cations do not change lattice sites, and that the charge balancing cations are in the nearby lattice sites of the same host phase. The lattice site must be of close size flexible enough to accommodate the transmuting cation. Better flexibility is characteristic to host phases with lattice sites having irregular coordination or are distorted, as shown in some examples below. The flexibility (solubility) of waste-form mineral phase(s) as hosts for a different valence substituted cation can be analyzed by performing coupled substitutions. When the number of cations changes during the substitution, a vacancy is either created or consumed, however the substitution must maintain electrical neutrality. These types of substitution are characteristic for polymorphic changes such as [47], where □ denotes a vacancy:

□ + Ba^2+^ → 2K^+^, or □ + Ca^2+^ → 2Na^+^, or □ + Na^+^ + 2Ca^2+^ → 3Na^+^ + Ca^2+^

In these coupled substitutions it is implicit that the exchanging cations occupy the same lattice sites, have the same coordination, and thus the crystallographic symmetry is maintained. These substitutions are typically written using Roman numerals that designate the number of oxygen atoms that coordinate around a given cation, e.g., ^VIII^Ca designates the octahedral VIII-fold coordination for the Ca^2+^ lattice site in oxyapatites:

$$\underset{host\;phase}{\underset{︸}{3{\mathrm{Ca}}^{2+}}}\to \underset{substituted\;phase}{\underset{︸}{2{\mathrm{Nd}}^{3+}\;+\;□}}$$

Calcium-neodymium-coupled substitutions were proven successful in the apatite (Ca_6_[SiO_4_]_3_) structure, resulting in a completely substituted Nd_4_□_2_[SiO_4_]_3_, where 2/3 of the lattice sites have Nd^3+^ and 1/3 are vacant [45,46,47]. Ca^2+^ is normally in VIII-fold coordination in the apatite and has a 1.12 Å atomic radius [47,48,49,50]. The Nd^3+^ cation in VIII-fold coordination also has an atomic radius of 1.11 Å [50], which is very close to the Ca^2+^ atomic radius in VIII-fold coordination. It has been shown that the rare earth elements from La^3+^ through Lu^3+^ can substitute for Ca^2+^ and form oxyapatites, RE_4.67_□_0.33_[SiO_4_]_3_O [51]. It was also shown [3] that even more complex but coupled substitutions were possible in the oxyapatite structure, such as:

$$\underset{host\;phase}{\underset{︸}{6^{VIII}{\mathrm{Ca}}^{2+}}}\to \underset{substituted\;phase}{\underset{︸}{1.7^{VIIII}{\mathrm{Nd}}^{3+}\;+\;1.7^{VIIII}Cs^{+}\;+\;0.86^{VIIII}{\mathrm{Ce}}^{4+}\;+\;0.86^{VIIII}{\mathrm{Sr}}^{2+}\;+\;0.88\;□}}$$

where the atomic radius, *r*, of Cs^+^ in VIII-fold coordination is 1.74 Å, Ce^4+^ in VIII-fold coordination is 0.97 Å, and Sr^2+^ in VIII-fold coordination is 1.26 Å. In this case small radii cations e.g., Ce^4+^ are mixed with larger radii cations such as Cs^+^, so that individual lattice sites can distort without perturbing the entire crystal structure of the host mineral. It should be noted that the exchanging cations are always in the same lattice site of the same host phase [3,45,46,51].

The substitutions such as those given above for the oxyapatites were also demonstrated to be possible in many other Ca-bearing mineral phases such as larnite (Ca_2_SiO_4_ or b-C_2_S), alite (calcium trisilicate or Ca_3_SiO_5_ or C_3_S), C_3_A (Ca_3_Al_2_O_6_) and C_4_AF (Ca_4_Al_2_Fe_2_O_10_), characteristic for cements [45,46]. This allowed Jantzen, et. al. [52,53] to make substitutions for Ca^2+^ in each phase (up to ~15 mole%) and prove possible the following additional substitutions:

$$\underset{host\;phase}{\underset{︸}{{\mathrm{Ca}}^{2+}+\;□}}\;\to \underset{substituted\;phase}{\underset{︸}{{2\mathrm{Cs}}^{+}}}$$

$$\underset{host\;phase}{\underset{︸}{{2\mathrm{Ca}}^{2+}+\;□}}\;\to \underset{substituted\;phase}{\underset{︸}{{\mathrm{Cs}}^{+}{\;+\;\mathrm{Sr}}_{0.5}^{2+}\;+{\;\mathrm{Nd}}_{0.17}^{3+}{\;+\;\mathrm{Ce}}_{0.25}^{4+}\;+\;0.08\;□}}$$

$$\underset{host\;phase}{\underset{︸}{1{.5\mathrm{Ca}}^{2+}+{\;\mathrm{Sr}}^{4+}}}\;\to \underset{substituted\;phase}{\underset{︸}{{\mathrm{Sr}}^{2+}\;+{\;\mathrm{Mo}}^{5+}\;+\;0.5\;□}}$$

$$\underset{host\;phase}{\underset{︸}{{4\mathrm{Ca}}^{2+}+{\;\mathrm{Fe}}^{3+}{\;+\;\mathrm{Al}}^{3+}}}\;\to \underset{substituted\;phase}{\underset{︸}{\;0{.66\mathrm{Nd}}^{3+}{\;+\;\mathrm{Zr}}^{4+}{\;+\;\mathrm{Mo}}^{4+}{\;+\;\mathrm{Sr}}^{2+}\;+{\;\mathrm{Ba}}^{2+}\;+\;1.33\;□}}$$

$$\underset{host\;phase}{\underset{︸}{\underset{r\sim 1.18\overset{\circ }{A}}{\underset{︸}{4^{IX}{\mathrm{Ca}}^{2+}}}+\;\underset{r=0.65\overset{\circ }{A}}{\underset{︸}{2^{VI}{\mathrm{Fe}}^{3+}}}}}\;\to \underset{substituted\;phase}{\underset{︸}{\;\underset{r=1.16\overset{\circ }{A}}{\underset{︸}{2.66^{IX}{\mathrm{Nd}}^{3+}}}\;+\;\underset{r=0.87\overset{\circ }{A}}{\underset{︸}{0.38^{VI}{\mathrm{Ce}}^{4+}}}\;+\;\underset{r=0.72\overset{\circ }{A}}{\underset{︸}{0.56^{VI}{\mathrm{Zr}}^{4+}}}\;+\;\underset{r=0.65\overset{\circ }{A}}{\underset{︸}{0.75^{VI}{\mathrm{Fe}}^{3+}}}\;+\;1.65\;□}}$$

It should be noted that the number of lattice sites have to be equivalent on the left-hand side and right hand site of the above equations.

These types of crystal-chemical substitutions have been studied in several waste-forms including SYNROC (SYNthetic ROCk) titanate phases containing zirconolite (CaZrTi_2_O_7_), perovskite (CaTiO_3_), and hollandites (nominally Ba(Al,Ti)_2_Ti_6_O_16_) [54], and in high alumina-tailored ceramic phases such as magnetoplumbites. Notable that magnetoplumbites were also found as a minor component of SYNROC, which immobilizes waste with high contents of Al [55].

Hollandite is the Cs^+^ host phase in the SYNROC phase assemblages. Its structure can be written as Ba_x_Cs_y_(Al,Fe)_2x+y_Ti_8-2xy_O_16_ where x + y must be <2 [56]. It has two types of octahedral sites, one of which accommodates trivalent cations like Al^3+^, Ti^3+^ and Fe^3+^, while the other accommodates Ti^4+^. The Cs^+^ is accommodated in tunnels that normally accommodate the Ba^2+^ cation, and Cs-Ba lattice sites are VIII-fold coordinated [54,56]. On synthesis the substitution orders and incommensurate superstructures result when Cs^+^ substitutes for Ba^2+^ [55]. Cs has been experimentally substituted for Ba when Fe^3+^ is substituted for Ti^4+^ in the VI-fold sites of hollandite. The species

CVIIIs0.28+BVIIIa1.002+︸A siteAVIl1.463+FVIe0.823+︸B siteTVIi5.724+︸C siteO16

has been synthesized by the sintering (frittage) of precursors in air at 1320 °C [56]. Ba–Al hollandite (Ba_1.16_Al_2.32_Ti_5.68_O_16_) was irradiated with 1–2.5 MeV electrons and β-irradiated up to summary doses of 4 × 10^8^ to 7 × 10^9^ Gy, after which it was found to contain Ti^3+^ centers and O_2_– superoxide ions that confirmed the mechanism of charge balance during transmutation [56]. Theoretically, the limiting value of Cs in hollandite is y = 0.81, which corresponds to a 9.54 wt% waste loading of Cs_2_O [57].

## 3. Synthesis of Ceramic Waste-forms

Research and development of ceramic materials based upon compounds on the base of the oxides and salt compositions were carried out for the immobilization of high-level wastes and the transmutation of minor actinides. Structures of such materials provide the incorporation of various cations and anions, either individually, or in various combinations and ratios. Structural forms in which can be implemented a wide isomorphism of cations and anions (including in different crystallographic positions) deserve special attention.

Among such structures the type NaZr_2_(PO_4_)_3_ (NZP) (analog—Mineral kosnarite) is regarded. NZP solid solutions may include more than half of the elements of Periodic Table of Elements in various combinations and ratios. The SYNROC developer calls them “near-universal solvent” [23], wherein this form of the consolidation of waste components is mono-phase in contrast to the multiphase SYNROC.

Ceramic materials are synthesized using the following methods: Pressing and sintering (frittage), hot isostatic or hot uniaxial pressing and other variants. Method Spark Plasma Sintering is the perspective for this aim. It provides a formation of virtually no porous ceramics having a relative density close to 99–100% for short time intervals (from 3 to 15 min). Reducing the porosity reduces the free surface, and therefore reduces the reaction surface and reactivity in heterogeneous systems with the participation of such materials. This in turn increases the heat, radiation and chemical stability of the ceramic.

Ceramic forms characteristics are presented here with their structures.

## 4. Crystalline Ceramic Phase:

### 4.1. Simple Oxides

**Figure 1:**
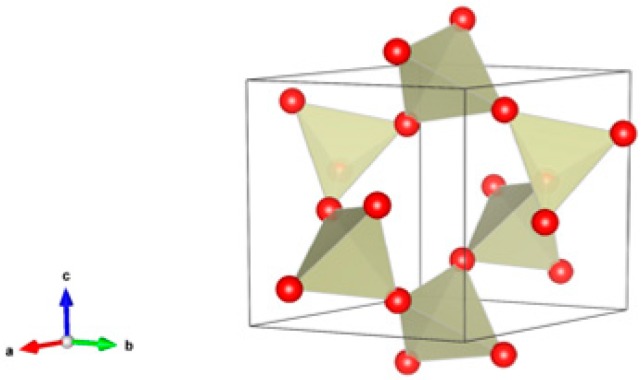
Silica, SiO_2_. α-quartz (low temperature modification), structure rhombohedra, Sp. gr. R3. β-quartz (high-temperature modification, it forms from α-quartz at 846 K, stable up to 1140 K). Structure hexagonal, Sp. gr. P6_2_22. Cations can be Li, Na, K. Mg, Ca, Mn, Cu, Ni, Pb B, Al, Fe, Cr, Ti, Zr and Te.

1. Silica, SiO_2_ [58,59,60,61,62,63,64,65,66,67,68,69,70,71,72,73,74,75], Figure 1.

Silicon dioxide, commonly known as silica (and/or quartz), is a prevalent element in the Earth’s crust, a mineral of most igneous and metamorphic rocks. The formula “SiO_2_” is commonly known as silicon dioxide. Silicon dioxide has a wide range of purposes, the main one being glass manufact-uring. In nature, silicon dioxide is commonly found as sand and quartz. Silica has polymorphism. It is stable under normal conditions of polymorphic modification—α-quartz (low temperature). Accordingly, β-quartz is called a high-temperature modification. Silica (α-quartz) possesses the rhombohedral structure, sp. gr. R3. Various elements with various oxidation states may attend in quartz: Li, Na, K. Mg, Ca, Mn, Cu, Ni, Pb B, Al, Fe, Cr, Ti, Zr and Te. Materials based on silicon oxide SiO_2_, Silica (quartz) were prepared in ceramic form by using methods: Hot isostatic pressing, laser sintering, cold pressing and sintering at 1500 °C, cold pressing and ultra-low temperature sintering at T = 554–600 °C (30 min) and Spark Plasma Sintering. 

Materials on the base of Silica can serve as a matrix for the immobilization of radioactive Iodine I-129 (half-life T_1/2_ = 15.7 × 10^3^ years).

**Figure 2:**
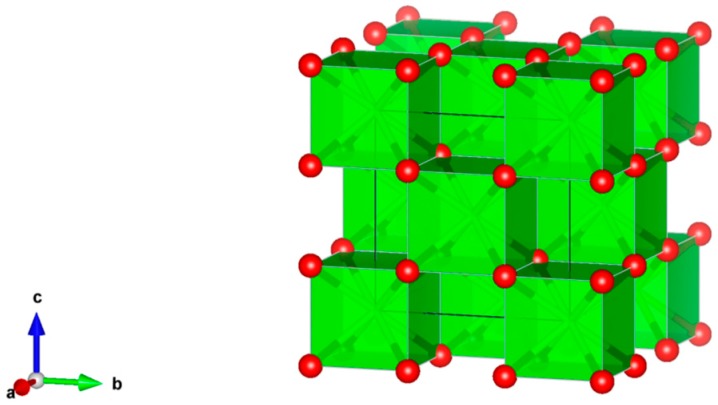
Fluorite, ZrO_2_. Structure cubic, Sp. gr. Fm3m. Cations can be Zr, Hf, Th, U, Np and Pu.

2. Oxides Fluorite, XO_2_ [76,77,78,79,80,81,82,83,84,85,86,87,88,89,90,91,92,93], Figure 2.

ZrO_2_, UO_2_, ThO_2_, HfO_2_, PuO_2_, α-U_2_O_3_ and Np_2_O_3_ have the simple fluorite cubic structure, sp. gr. Fm3m. Fluorite has physical properties that allow it to be used for a wide variety of chemical, metallurgical and ceramic processes. The waste ceramics with high zirconia and alumina contents, and Y_2_O_3_-stabilized zirconia with fluorite structure, are the main host phases for actinide, rare earth elements, as well as Cs, Sr in high-level radioactive waste (HLW). Ceramics were made by HIP, HUP, press and sinter, melting and crystallization and by Spark Plasma Sintering with high relative density (up to 97–99%).

### 4.2. Complex Oxides

**Figure 3:**
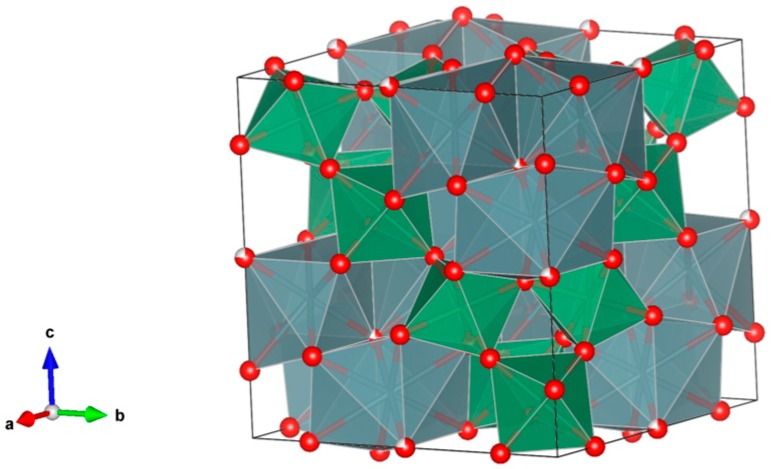
Pyrochlore. A_2_B_2_O_7_. Structure cubic, Sp. gr. Fd3m. A-site-cations can be Na, Ca, Y, lanthanides, Th and U, while on the B-site—cations can be Fe^3+^, Ti, Zr, Nb and Ta.

3. Pyrochlore [86,94,95,96,97,98,99,100,101,102,103,104,105,106,107,108,109,110,111,112,113,114,115,116,117], Figure 3.

Many compounds with A_2_B_2_O_7_ stoichiometry adopt the pyrochlore structure. A derivative of the fluorite structure type, A_2_B_2_O_7_, where the A-site contains large cations (Na, Ca, U, Th, Y and lanthanides) and the B-site contains smaller, higher valence cations (Nb, Ta, Ti, Zr and Fe^3+^). Structure: Cubic, Sp. gr. $Fd_{\bar{3}}$m, z = 8. Ceramics were prepared by cold pressing and sintering.

**Figure 4:**
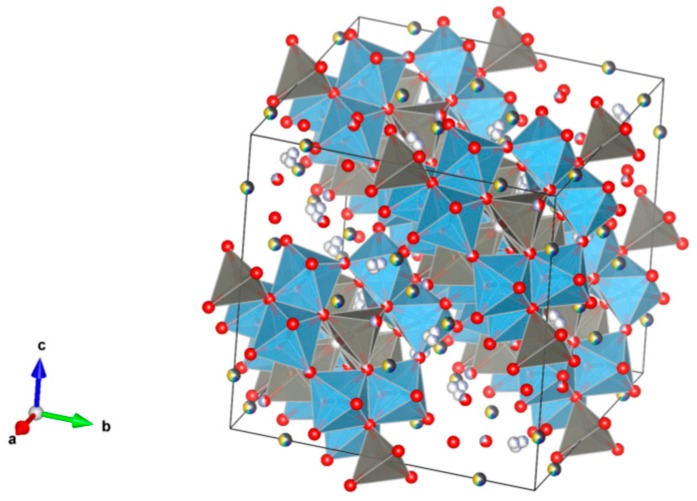
Murataite. A_6_B_12_C_5_TX_40-x_. Structure: Cubic, Sp. gr. F4m. Cations can be U, Np, Pu, Am, Cm and REE, including Gd (a neutron absorber).

4. Murataite [104,106,108,118,119,120,121,122,123,124,125,126,127,128,129,130,131], Figure 4.

Murataite is a derivative of the isometric fluorite structure A_6_B_12_C_5_TX_40-x_, with multiple units of the fluorite unit cell; hosts U, Np, Pu, Am, Cm and REE, including Gd, a neutron absorber. It forms in solid solution with pyrochlore. Structure: Cubic, Sp. gr. $F4_{\bar{3}}$m, z = 4. Ceramics were prepared by cold pressing and sintering.

**Figure 5:**
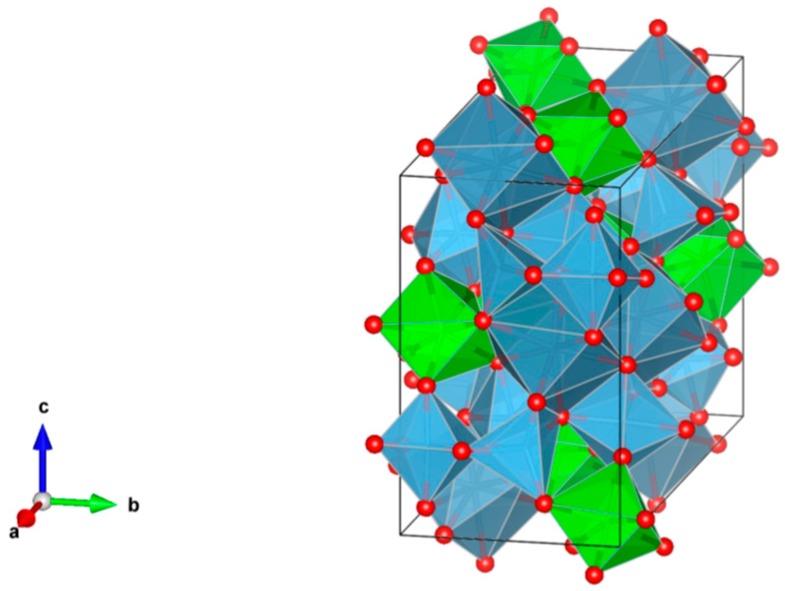
Zirconolite. CaZrTi_2_O_7_, Structure monoclinic, Sp. gr. C2/c. Cations can be Gd, Hf, Ce, Th, U, Pu and Nb.

5. Zirconolite [112,113,132,133,134,135,136,137,138,139,140,141,142,143,144,145,146,147,148,149,150], Figure 5.

Monoclinic CaZrTi_2_O_7_, has a fluorite-derived structure closely related to pyrochlore, where Gd, Hf, Ce, Th, U, Pu and Nb may be accommodated on the Ca/Zr-sites, as in the case of Ca(Zr,Pu)Ti_2_O_7_. Structure: Trigon., Pr. gr. C2/c. Ceramics were prepared by cold pressing and sintering.

**Figure 6:**
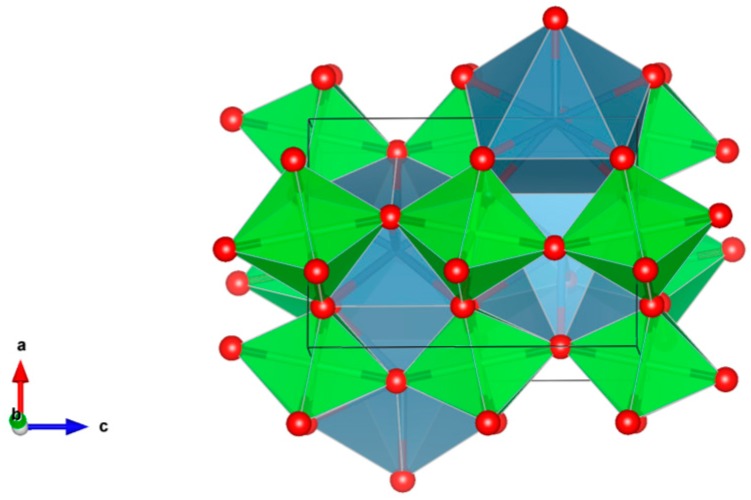
Perovskite. CaTiO_3_, Structure cubic, Sp. gr. Pm3m. Cations can be Ca, Y, REE, Ti, Zr, U and Pu.

6. Perovskite [110,134,140,151,152,153,154,155,156,157,158,159], Figure 6.

CaTiO_3_ has a wide range of compositions as stable solid-solutions; orthorhombic; consists of a 3-dimensional network of corner-sharing TiO_6_ octahedra, with Ca occupying the large void spaces between the octahedra (the corner-sharing octahedra are located on the eight corners of a slightly distorted cube). Plutonium, other actinides and rare-earth elements can occupy the Ca site in the structure, as in (Ca,Pu)TiO_3_. The octahedra can also tilt to accommodate larger cations in the Ca site. Structure: Cubic, sp. gr. Pm3m; rombohedral, Sp. gr. Pnma; may include: Ca, Y, REE, Ti, Zr, U and Pu. Ceramics were prepared by cold pressing and sintering, and by hot pressing enabling densities up to 90–98% of theoretical.

**Figure 7:**
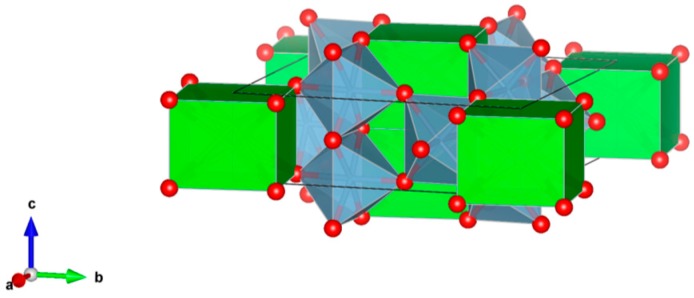
Hollandite. Ba_1.2_(Al,Ti)_8_O_16_. Structure tetragon, Sp. gr. I4/m, monocl, Sp. gr. I2/m. Cations can be Na, K, Cs, Mg, Ca, Sr, Ba, Al, Fe, Mn^3+^, Si, Ti and Mn^4+^.

7. Hollandite [160,161,162,163,164,165,166,167,168,169], Figure 7.

Ba_1.2_(Al,Ti)_8_O_16_ tunnels between TiO_6_ octahedra to accommodate ^133^Ba, ^137^Cs and ^90^Sr. Structure: Tetragon, Sp. gr. I4/m, Z = 4 and monocl., Sp. gr. I2/m, z = 1; may include: Na, K, Cs, Mg, Ca, Ba, Al, Fe, Mn^3+^, Si, Ti and Mn^4+^. Ceramics were prepared by cold pressing and sintering.

**Figure 8:**
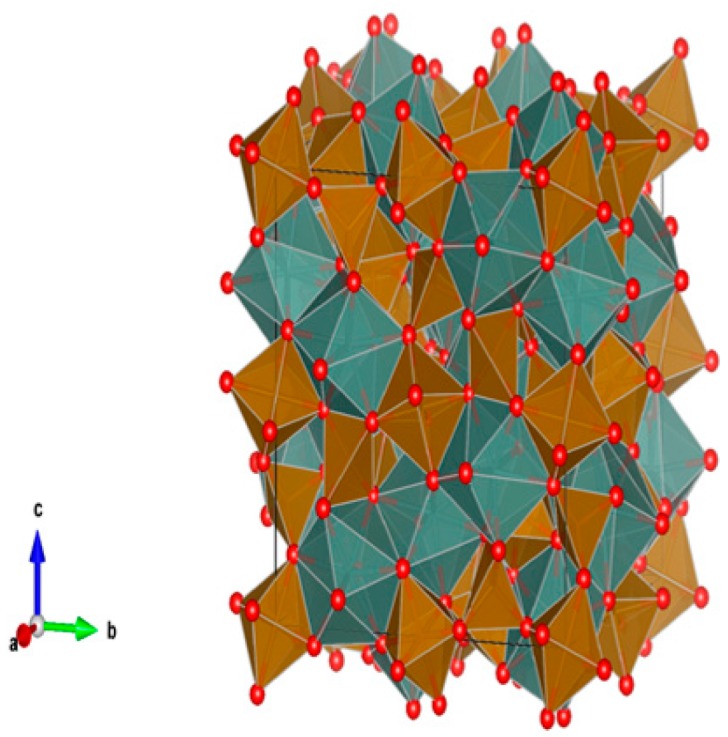
Garnet, Ca_3_Al_2_Si_3_O_12_. Structure cubic, Sp. gr. Ia3d. Cations can be Mg, Ca, Mn, Co, Cd, Al, Sc, Fe, Ga, Y, In, La, REE, Ti, Zr, Ru, Sn, N, P, V and As.

**Figure 9:**
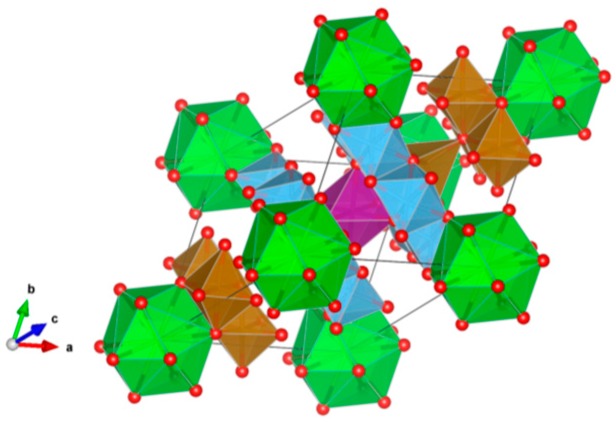
Crichtonite. Sr(Mn,Y,U)Fe_2_(Ti,Fe,Cr,V)_18_(O,OH)_38_. Structure rombohedral, Sp. gr. R3. Cations can be Mg, Mn, Ni, Cu, Mn, Sr, Pb, Cr, Fe, Y, La, Ce, Ti, Zr, Hf, U, V and Nb.

8. Garnet [87,89,104,105,170,171,172,173,174,175,176,177,178,179,180,181,182,183,184,185,186,187,188,189,190,191,192,193,194], Figure 8.

(1) ^[8]^A_3_^[6]^B_2_[TiO_4_]_3_, e.g., ^[8]^(Ca,Gd, actinides)^[6]^Fe_2_^[4]^Fe_3_O_12_.
(2) A_3_B_2_(XO_4_)_3_; distorted cubic structure; BO_6_ octahedra and XO_4_ tetrahedra establish a framework structure alternately sharing corners; A and B sites can host actinides, REs, Y, Mg, Ca, Fe^2+^, Mn^2+^ and X = Cr^3+^, Fe^3+^, Al^3+^, Ga^3+^, Si^4+^, Ge^4+^ and V^5+^. Structure: Cubic, Sp. gr. Ia3d, z = 8. Ceramics were prepared by cold pressing and sintering and using Spark Plasma Sintering with high relative density up to 98–99% of theoretical.

9. Crichtonite [131,195,196,197,198,199,200,201,202], Figure 9.

(Sr,Pb,La,Ce,Y)(Ti,Fe^3+^,Mn,Mg,Zn,Cr,Al,Zr,Hf,U,V,Nb,Sn,Cu,Ni)_21_O_38_. Sr, La, Ce, Y positions are indicated by the solid circles. Other cations are in the octahedral positions. Structure: Rombohedral, Sp. gr. R3. Ceramics were prepared by hot pressing.

**Figure 10:**
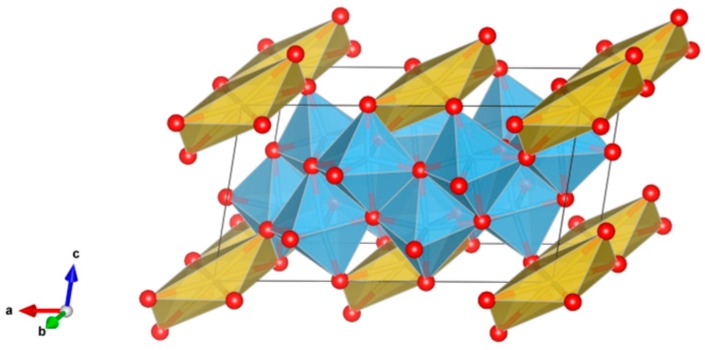
Freudenbergite (spinel). Na_2_Al_2_(Ti,Fe)_6_O_16_;. Structure monocl. Sp. gr. C12/m1. Cations can be Na, K, Mg, Co, Ni, Zn, Al, Ti^3+^, Cr, Fe, Ga, Si and Nb.

10. Freudenbergite [153,155,203,204], Figure 10.

Na_2_Al_2_(Ti,Fe)_6_O_16_ a spinel-based phase suitable for incorporating Al-rich wastes from Al fuel cladding/decladding. The A site can accommodate Na and K while the different octahedral sites can accommodate Mg, Co, Ni, Zn, Al, Ti^3+^, Cr, Fe, Ga, Si and Nb. Structure: Monocl., Sp. gr. C12/m1. Ceramics were prepared by cold pressing and sintering, ρ = 90%.

**Figure 11:**
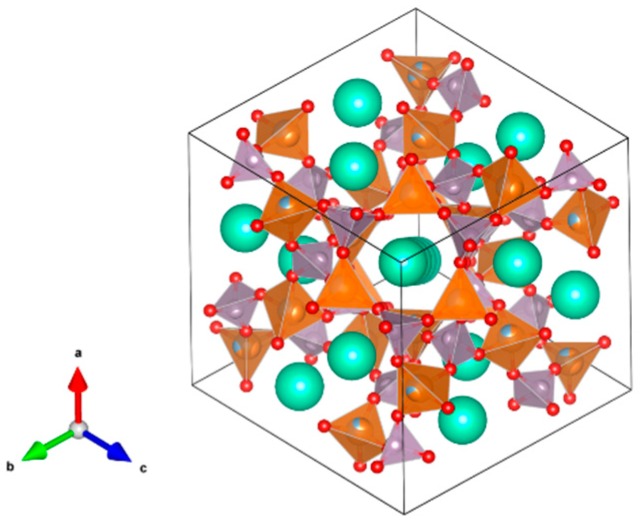
P-Pollucite. (Na,K,Rb,Cs)MgAl_0.5_P_1.5_O_6_; Structure cubic, Sp. gr. I4_1_32. Cations can be Li, Na, K, Rb, Cs, Tl, Be, Mg, Mn, Co, Ni, Cd, Sr, Ba, Sr. Ba, B, Al, Fe, Si, Ti, P, V, Nb and Ta.

11. P-Pollucite [205,206,207,208,209,210,211,212,213,214,215], Figure 11.

The ability of the pollucite structure to include large 1-, 2- and 3-valent cations allows flexibility to select the desired model composition. When replacing the cations it will be becomes possible to use cheap components; the introduction of small cations increases the concentration of cesium in the composition of the mono-phase product. Structure: Cubic, sp. gr. I4_1_32, z = 16; may include: Li, Na, K, Rb, Cs, Tl, Be, Mg, Sr, Ba, Cd, Mn, Co, Ni, Cu, Zn, B, Al, Fe, Si, Ti, P, V, Nb and Ta. Compounds are hydrolytically and radiation-wise stable. Ceramics were prepared by cold pressing and sintering and Spark Plasma Sintering with high relative density (at last those up to 98–99%).

**Figure 12:**
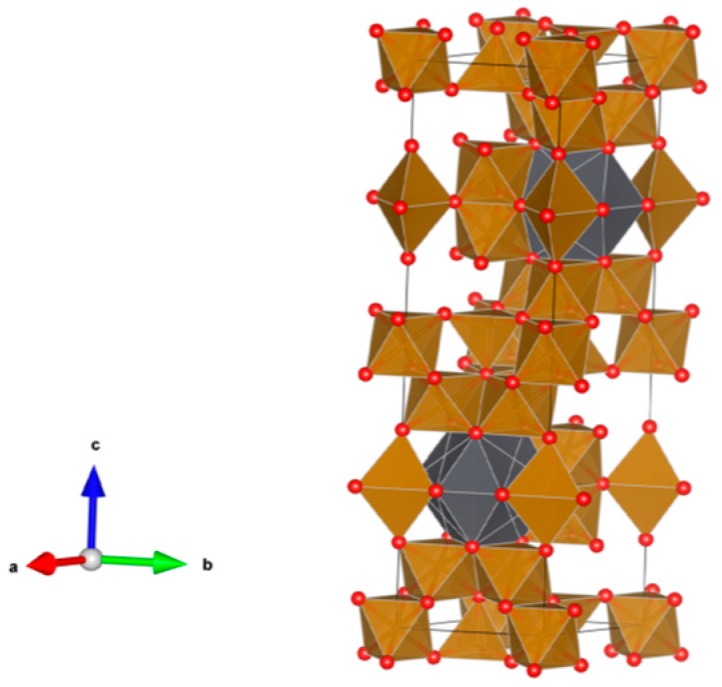
Magnetoplumbite. (Sr,Ba, ((Na,Cs)_0.5_+La_0.5_))(Al,Fe)_12_O_19_. Structure hexagon., Sp. gr. P63/mmc. Cations can be Na, Cs, Mg, Sr, Ba, Pb, Mn, Co, Cu, Al, Fe, Sc, Y, La, Ce, Sm, Gd, Yb, Lu, An, Si, Ti and Sn.

12. Magnetoplumbites (aluminates) [13,55,216,217,218,219,220,221,222,223,224], Figure 12.

Nominally X(Al,Fe)_12_O_19_, where X = Sr, Ba, (Cs_0.5_ + La_0.5_) and (Na_0.5_ + La_0.5_). The X site is XII-fold coordinated and both Cs^+^/Ba^2+^-Fe^3+^/Fe^2+^ or Cs^+^/Ba^2+^-Ti^4+^/Ti^3+^ type substitutions can occur. Accommodating structures because they are composed of spinel blocks with both IV-fold and VI-fold coordinated sites for multivalent cations, and interspinel layers which have unusual V-fold sites for small cations. The interspinel layers also accommodate large cations of 1.15–1.84 Å, replacing oxygen in XII-fold sites in the anion close packed structure. The large ions may be monovalent, divalent, or trivalent with balancing charge substitutions either in the interspinel layer (Na_0.5_ + La_0.5_) or between the interspinel layer and the spinel blocks (Cs^+^/Ba^2+^–Fe^3+^/Fe^2+^ or Cs^+^/Ba^2+^–Ti^4+^/Ti^3+^). Structure: Hexagon., Sp. gr. P6_3_/mmc, z = 2; may include: Na, Cs, Mg, Sr, Ba, Pb, Mn, Co, Cu, Al, Fe, Sc, Y, La, Ce, Sm, Gd, Yb, Lu, actinides, Si, Ti and Sn. Ceramics were prepared by cold pressing and sintering and by hot pressing.

**Figure 13:**
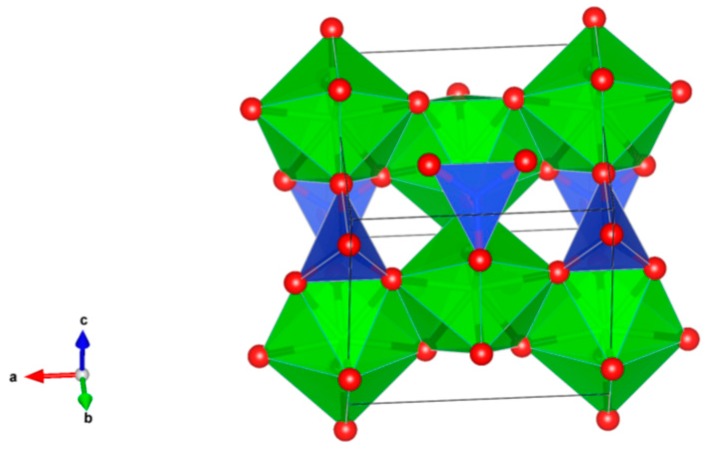
Zircon/Thorite/Coffinite. ZrSiO_4_/ThSiO_4_/USiO_4_. Structure tetragon., Sp. gr. I41/amd. Cations can be Na, Tl, Mg, Ca, Mn, Co, Fe, Ti, REE, Ti, Th, U, Pi, P, V, Mo and Se.

13. Zircon/Thorite/Coffinite [83,110,140,225,226,227,228,229,230,231,232,233,234,235], Figure 13.

ZrSiO_4_/ThSiO_4_/USiO_4_; zircon is an extremely durable mineral that is commonly used for U/Pb age-dating, as high uranium concentrations (up to 20,000 ppm) may be present; the PuSiO_4_ end member is known, and Ce, Hf and Gd have been found to substitute for Zr. Structure: Tetragon. Sp. gr. I41/amd, z = 4; may include: REE, Th, U, Pu; Na, Mg, Ca, Mn, Co, Fe, Ti, P, V, Se and Mo. Ceramics were prepared by hot pressing, ρ = 99.1% and by Spark Plasma Sintering, ρ = 99%

**Figure 14:**
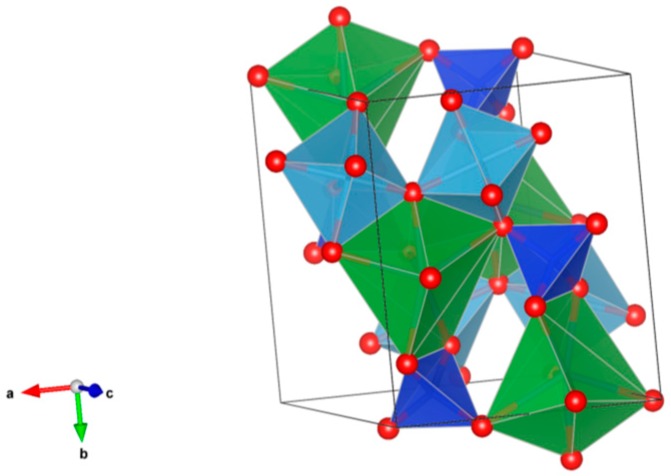
Titanite (sphene). CaTiSiO_5_ [CaTiO(SiO_4_)]. Structure monocl., Sp. gr. P_2_I/a. Cations can be Mg, Ca, Sr, Ba, Mn, Al, Fe, Cr, Ce, Y, Zr, Th and F.

14. Titanite (sphene) [104,110,236,237,238], Figure 14.

CaTiSiO_5_ [CaTiO(SiO_4_)]. Structure: Monocl. Sp. gr. P_2_I/a, Z = 4; may include: Mg, Ca, Sr, Ba, Mn, Al, Fe, Cr, Ce, Y, Zr, Th and F. Ceramics are known as a matrix for actinide immobilization, and were prepared by cold pressing and sintering.

**Figure 15:**
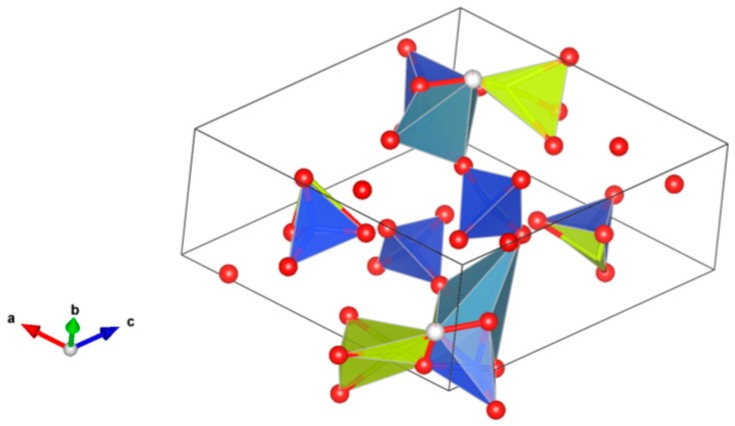
Britholite (silicate apatite, oxy-apatite). (REE,Ca)_5_(SiO_4_,PO_4_)_3_(OH,F)-Structure monoclin. Sp. gr. P2_1_/hexagonal, Sp. gr. P6_3_/m. Cations can be Cs, Sr, B, REE, Th, U, Np and Pu.

15. Britholite (silicate apatite; also known as oxy-apatite in the literature) [3,46,51,239,240,241,242,243,244,245,246,247,248,249], Figure 15.

(REE,Ca)_5_(SiO_4_,PO_4_)_3_(OH,F); i.e., Ca_2_Nd_8_(SiO_4_)_6_O_2_, Ca_2_La_8_(SiO_4_)_6_O_2_; based on ionic radii of Nd^3+^, La^3+^ and Pu^3+^, an extensive range of solubility for Pu^3+^ substitution for the Nd or La, particularly on the *6h* site, is expected. Since there is an extensive range in the Ca/RE ratio in these silicate apatites, a fair amount of Pu^4+^ substitution may be possible; La^3+^ through Lu^3+^ can substitute for Ca^2+^ and form oxyapatites, RE_4.67_□_0.33_[SiO_4_]_3_O; can also accommodate Cs, Sr, B, Th, U and Np. Structure: Monocl., Sp. gr. P2_1_ and hexagon. Sp. gr. P6_3_/m. Ceramics were prepared by cold pressing and sintering, ρ = 95%.

### 4.3. Framework Silicates

**Figure 16:**
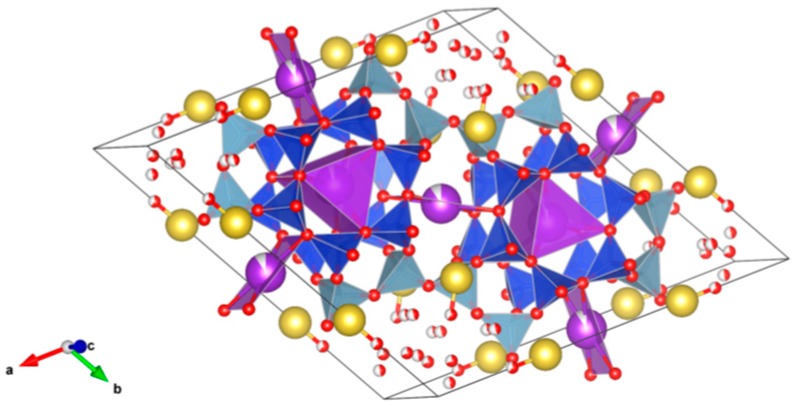
Zeolites. X_x/n_[(AlO_2_)_x_(SiO_2_)_y_] (where Xn+ is the charge balancing counter-ion). Structure depends on chemical composition. Cations can be Na, K, NH^4+^, Cs, Mg, Ca, Sr, Co, Fe, Ga, REE and Ti.

16. Zeolites [75,250,251,252,253,254,255,256,257,258,259,260,261,262,263,264,265,266], Figure 16.

(X_x/n_[(AlO_2_)_x_(SiO_2_)_y_] where X is the charge balancing counter-ion, n is the charge of the counter-ion, x is the number of charge-deficient alumina sites, and y is the number of charge-neutral silica sites. Zeolites are characterized by internal voids, channels, pores, and/or cavities of well-defined size in the nanometer range, ≈4–13 Å. The channels and/or cavities may be occupied by charge compensating ions and water molecules. Zeolites like Ag-Mordenite selectively sorbs I_2_ (^129^I); certain zeolites can be converted to condensed oxide ceramics by heating. This process is particularly attractive for waste-form synthesis because contaminants capture and immobilization is performed with minimal steps. Structure of Zeolite-A showing alternate Al and Si atom ordering but omitting the tetrahedral oxygens around each Al and Si may include Na, K, NH_4_^+^, Cs, Mg, Ca, Sr, Co, Fe, Ga, REE and Ti. 45 natural zeolites and 100 artificial ones are known. Ceramics were prepared by hot pressing.

**Figure 17:**
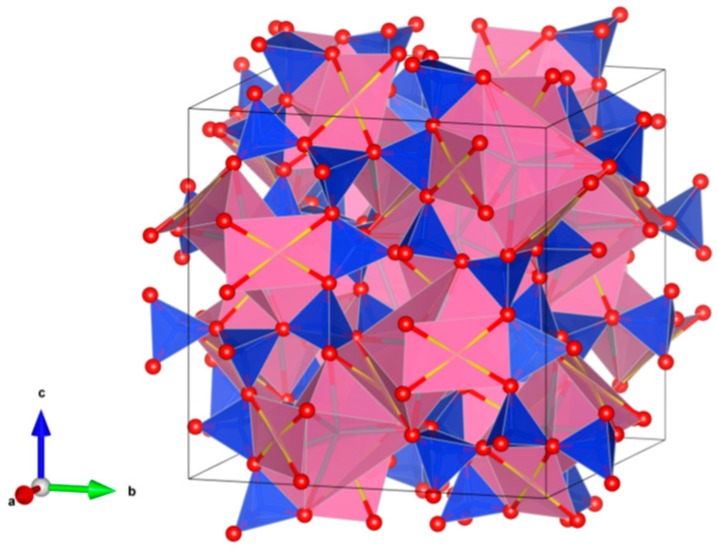
Pollucite. (Ca,Na)_2_Al_2_Si_4_O_12_·2H_2_O. Structure cubic, Sp. gr. Ia3d. Cations can be Li, Na, K, Rb, Cs, Tl, Be, Mg, Sr, Ba, Cd, Mn, Co, Ni, Cu, Zn, B, Al, Fe, Si, Ti, P, V and Nb.

17. Pollucite [37,87,212,214,215,259,267,268,269,270,271,272,273,274,275,276,277,278,279,280,281,282,283,284,285,286,287,288,289,290,291,292,293], Figure 17.

(Ca,Na)_2_Al_2_Si_4_O_12_·2H_2_O; host for fission products such as ^137^Cs. Structure: Cubic, Sp. gr. Ia3d, z = 16; may include: Li, Na, K, Rb, Cs, Tl, Be, Mg, Sr, Ba, Cd, Mn, Co, Ni, Cu, Zn, B, Al, Fe, Si, Ti, P, V and Nb. Ceramics were prepared by Spark Plasma Sintering with high relative density (up to 96%).

**Figure 18:**
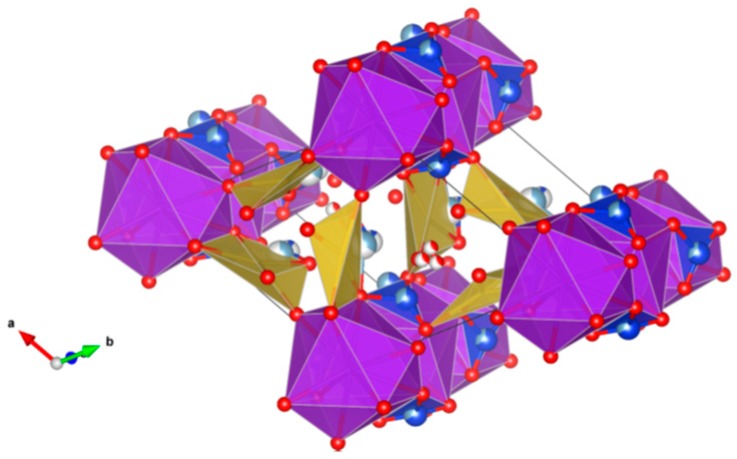
Nepheline/Leucite. (Na, K)AlSiO_4_/K[AlSi_2_O_6_]. Structure hexagon., Sp. gr. P6_3_/tetragonal, Sp. gr. I4_1_/a and I4_1_/acd or cubic, Sp. gr. Ia3d. Cations can be Li, Na, K, Rb, Cs, Be, Mg, Ca, Ba, Pb, Mn, Co, Ni Al, Fe, Cr, Si, Ti and V.

18. Nepheline/Leucite [37,58,61,73,155,294,295,296,297], Figure 18.

NaAlSiO_4_ silica “stuffed derivative” ring type structure; some polymorphs have large nine-fold cation cage sites, while others have 12-fold cage-like voids that can hold large cations (Cs, K, Ca). Natural nepheline structure accommodates Fe, Ti and Mg. Two-dimensional representation of the structure of nepheline showing the smaller 8 oxygen sites that are occupied by Na and the larger 9 oxygen sites that are occupied by K and larger ions, such as Cs and Ca. Structure may include: Li, Na, K, Rb, Cs, Be, Mg, Ca, Ba, Pb, Mn, Co, Ni, Al, Fe, Cr, Si, Ti and V. Structure: Hexagon. Sp. gr. P6_3_, z = 2. Leucite. Structure: Tetragon. Sp. gr. I4_1_/a and I4_1_/acd; cubic, Sp. gr. Ia3d, z = 16.

**Figure 19:**
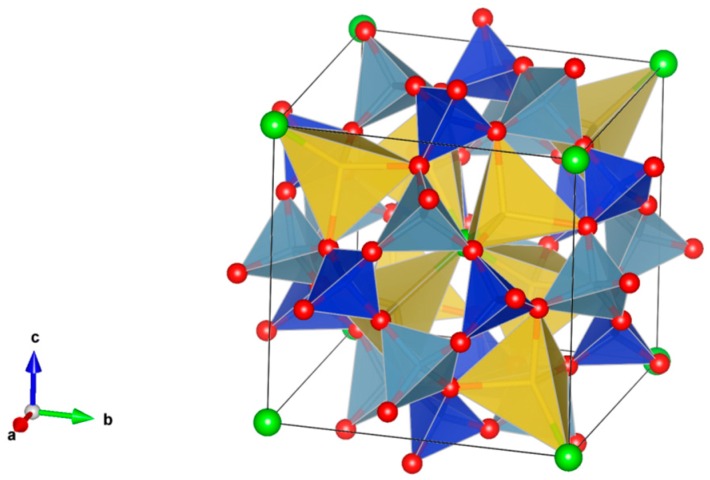
Sodalite.group minerals. Sodalite/Nosean/Hauyne/Helvite/Danalite/Genthelvite/Lazurite. (Na,K)_6_[Al_6_Si_6_O_24_]·(2NaCl)/(Na,K)_6_[Al_6_Si_6_O_24_](Na_2_SO_4_)/(Na)_6_[Al_6_Si_6_O_24_]((Ca,Na)SO_4_)_1-2_/(Mn_4_[Be_3_Si_3_O_12_]S/(Fe_4_[Be_3_Si_3_O_12_]S)/(Zn_4_[Be_3_Si_3_O_12_]S)/(Ca,Na)_6_[Al_6_Si_6_O_24_]((Ca,Na),S,SO_4_,Cl)_x_; Structure cubic, Sp. gr. P3n Cations and anions can be Na, K, Be, Mg, Ca, Mn, Fe, Al, Si, Ti, Cl, SO_4_ and CO_3_.

**Figure 20:**
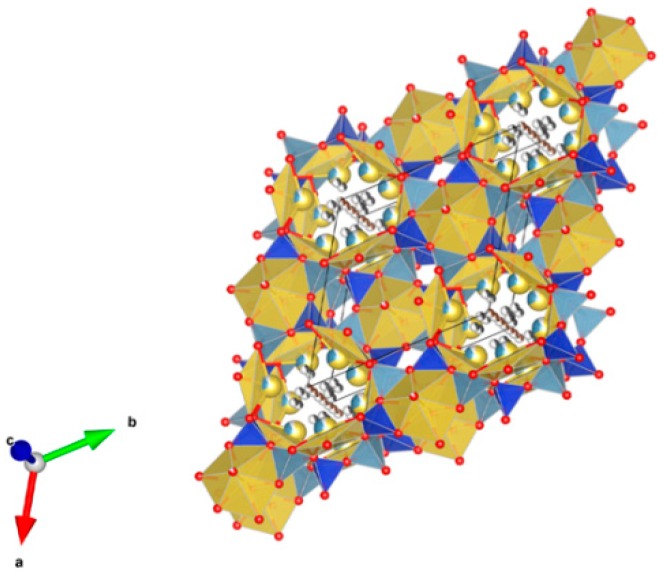
Cancrinite. (Na,Ca,K)_6_[Al_6_Si_6_O_24_]((Na,Ca,K)_2_CO_3_)_1.6_·2.1H_2_O. Structure hexagonal, Sp. gr. P6_3._ Cations and anions can be Na, K, Ca. Al, Si, SO_4_ and Cl.

19. Sodalite Group (name of mineral changes with anions sequestered in cage structure) [37,264,295,298,299,300,301,302,303,304,305,306,307,308,309,310,311,312,313], Figure 19.

(1) Sodalite Na_8_Cl_2_Al_6_Si_6_O_24_, also written as (Na,K)_6_[Al_6_Si_6_O_24_]·(2NaCl) to demonstrate that 2Cl and associated Na atoms are in a cage structure defined by the aluminosilicate tetrahedra of six adjoining NaAlSiO_4_, is a naturally occurring feldspathoid mineral. It incorporates alkali, alkaline earths, rare earth elements, halide fission products and trace quantities of U and Pu. Sodalite was and it is being investigated as a durable host for the waste generated from electro-refining operations deployed for the reprocessing of metal fuel. Supercalcines which are high temperature, silicate-based “natural mineral” assemblages proposed for HLW waste stabilization in the United States in 1973–1985, contained sodalites as minor phases retaining Cs, Sr and Mo, e.g., Na_6_[Al_6_Si_6_O_24_](NaMoO_4_)_2_. Sodalite structures are known to retain B, Ge, I, Br and Re in the cage-like structures. Structure of Sodalite showing (a) two-dimensional projection of the (b) three-dimensional structure and (c) the four fold ionic coordination of the Na site to the Cl-ion and three framework oxygen bonds. Structure: Cubic, Sp. gr. $P_{\bar{4}}3n$, z = 1; may include: Na, K, Mg, Ca, Mn, Fe, Al, Si, Ti, Cl, SO_4_ and CO_3_. Ceramics were prepared by cold pressing and sintering; by HIP.
(2) Nosean, (Na,K)_6_[Al_6_Si_6_O_24_](Na_2_SO_4_)), silica “stuffed derivative” sodalite cage type structure host mineral for sulfate or sulfide species.
(3) Hauyne, (Na)_6_[Al_6_Si_6_O_24_]((Ca,Na)SO_4_)_1-2_ sodalite family; can accommodate either Na_2_SO_4_ or CaSO_4_.
(4) Helvite (Mn_4_[Be_3_Si_3_O_12_]S: Be (beryllium) can be substituted in place of Al and S_2_ in the cage structure along with Fe, Mn and Zn.
(5) Danalite (Fe_4_[Be_3_Si_3_O_12_]S).
(6) Genthelvite (Zn_4_[Be_3_Si_3_O_12_]S).
(7) Lazurite, (Ca,Na)_6_[Al_6_Si_6_O_24_]((Ca,Na)S,SO_4_,Cl)_x_; can accommodate either SO_4_ or S_2_, Ca or Na and Cl.

20. Cancrinite [37,314,315,316,317,318,319], Figure 20.

Cancrinite is a complex carbonate and silicate of sodium, calcium and aluminum with the formula (Na,Ca,K)_6_[Al_6_Si_6_O_24_](( Na,Ca,K)_2_CO_3_)_1.6_·2.1H_2_O. It is classed as a member of the feldspathoid group of minerals. Cancrinite is unusual in that it is one of the few silicate minerals to have a carbonate ion (CO_3_^2−^) present in its structure. Mineral cancrinite will also contain some percentages of sulfate ions (SO_4_^2−^) and a chlorine ion (Cl^−^). Structure: Hexagonal, Sp. gr. P6_3_.

**Figure 21:**
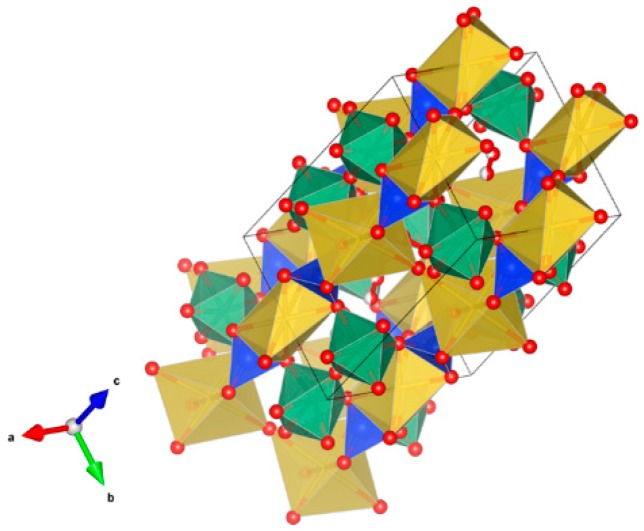
SilicoTitanate (CST). SiTiO_4_. Structure cubic, Sp. gr. Pm3m up to 105 °C, after-tetragonal Sp. gr. I4/mcm or P4_2_/mcm. Cations can be Na, K, Cs, Ca, Sr, Ba, Pb, Al, REE, Bi, Ti, Zr, Nb and Ta.

21. Crystalline SilicoTitanate (CST) [73,110,273,274,275,277,320,321,322,323,324], Figure 21.

[(Ca,N2a,K,Ba)AlSiO_4_ incorporates Na, K, Cs, Ca, Sr, Ba, Pb, Al, REE, Bi, Ti, Zr, Nb and Ta. Crystal structure of Cs exchanged Nb–titanium silicate. Structure: Cubic, sp. gr. Pm3m up to 105 °C, after tetragon. Sp. gr. I4/mcm or P4_2_/mcm. Ceramics were prepared by hot isostatic pressing.

**Figure 22:**
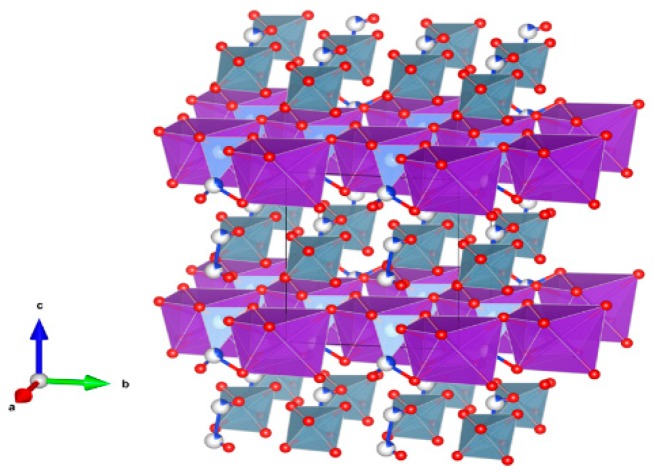
Micas (Dehydroxylated). XY_2–3_Z_4_O_10_(OH, F)_2_ with X = K, Na, Ba, Ca, Cs, (H_3_O) and (NH_4_); Y = Al, Mg, Fe^2+^, Li, Cr, Mn, V and Zn; and Z = Si, Al, Fe^3+^, Be and Ti. Structure monoclinic, Sp. gr. C2/c.

22. Micas (Dehydroxylated) [37,325,326,327,328,329,330], Figure 22.

The following dehydroxylated micas have been synthesized phase pure: LiAl_3_Si_3_O_11_, NaAl_3_Si_3_O_11_, KAl_3_Si_3_O_11_, RbAl_3_Si_3_O_11_, CsAl_3_Si_3_O_11_, TlAl_3_Si_3_O_11_, Ca_0.5_□_0.5_Al_3_Si_3_O_11_, Sr_0.5_□_0.5_Al_3_Si_3_O_11_, Ba_0.5_□_0.5_Al_3_Si_3_O_11_ and La_0.33_□_0.66_Al_3_Si_3_O_11_. In the Cs mica up to 30 wt% Cs_2_O can be accommodated, in the Rb-mica up to 22 wt% Rb_2_O can be accommodated, and in the Ba-mica up to 19 wt% BaO can be accommodated. Mg, Fe^2+^, Fe^3+^, Mn, Li, Cr, Ti and V can substitute for VI-fold coordinated Al^3+^. Structure: Monoclinic. Sp. gr. C2/c.

### 4.4. Phosphates

**Figure 23:**
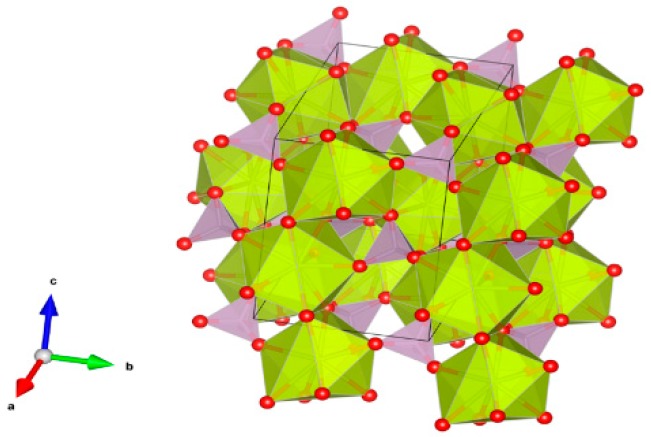
Monazite. (Ce,La,Nd,Th)(PO_4_,SiO_4_). Structure monoclinic, Sp. gr. P21/n. Cations can be Li, Na, K, Rb, Mg, Ca, Sr, Ba, Cd, Pb, Bi, Y, La, Pr, Nd, Sm, Eu, Gd, Tb, Yb, Am, Cm, Cf, Es, Ge, Zr, Th, U, Np, Pu, Cm; Si, Se, V, As and S.

23. Monazite [12,16,17,18,87,89,140,141,231,235,244,293,331,332,333,334,335,336,337,338,339,340,341,342,343,344,345,346,347,348,349,350,351,352,353,354,355,356,357,358,359], Figure 23.

CePO_4_ or LaPO_4_ are corrosion-resistant materials and can incorporate a large range of radionuclides including actinides and toxic metals into its structure. Monazite was proposed as a potential host phase for excess weapons plutonium and radionuclides, and toxic metals in glass ceramic waste-forms for low-level and hazardous wastes. Monazite structure (monazite mineral CePO_4_) has wide capacity isomorphous through which the cerium and phosphorus can be substituted for other elements, e.g.,: Ce → Li, Na, K, Rb, Mg, Ca, Sr, Ba, Cd, Pb, Bi, Y, La, Pr, Nd, Sm, Eu, Gd, Tb, Yb, Am, Cm, Cf, Es, Ge, Zr, Th, Np, U and Pu; P → Cr, Si, Se, V, As and S. Alternating chains of PO_4_ tetrahedra and REO_9_ polyhedra. Structure: Monoclinic. Sp. gr. P2_1_/n. Ceramics were prepared by cold pressing and sintering (ρ = 90–95%), hot pressing (ρ = 97%) and Spark Plasma Sintering with high relative density (up to 98–99%).

**Figure 24:**
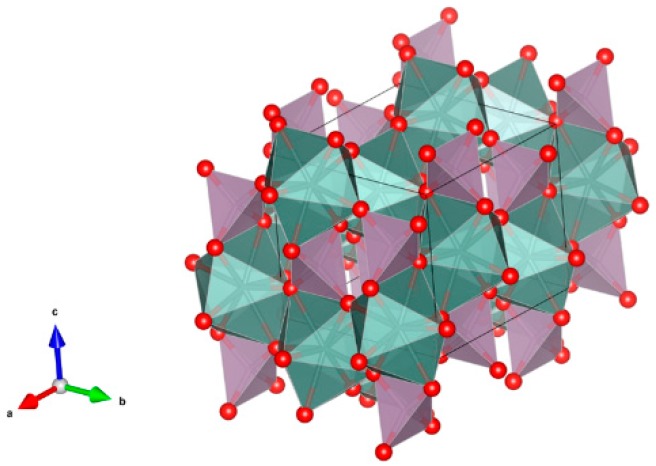
Xenotime (YPO_4_). Ce,La,Nd,Th)(PO_4_,SiO_4_). Structure tetragonal, Sp. gr. I41/amd. Cations can be Be, Ca, Al, Sc, La, Ce, Er, Dy–Lu, Zr, Th and U.

24. Xenotime [231,334,344,360,361,362,363], Figure 24.

YPO_4_. Structure: Tetragonal. Sp.gr. I4_1_/amd, z = 4, C.N.Y-O_n_, n = 8. Isomorph including: Be, Ca, Al, Sc, La, Ce, Er, Dy–Lu, Zr, Th and U. Ceramics were prepared by cold pressing and sintering.

**Figure 25:**
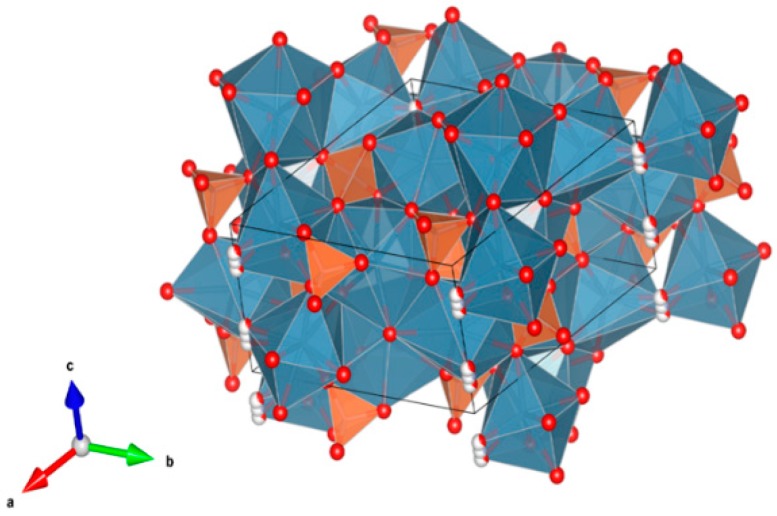
Apatite. Ca_5_(PO_4_)_3_(OH,F,Cl). Apatite. Structure hexagonal, Sp. gr. P63/m, monoclinic, Sp. gr. P21/b. Cations and anions can be Na, K, Cs, Mg, Ca, Mn, Ni, Sr, Ba, Cd, Hg, Pb, Cr, Y, REE, Cm, Si, Th, U, P, V, As, S, F, Cl, OH and CO_3_.

25. Apatite [3,37,87,240,241,332,364,365,366,367,368,369,370,371,372,373,374,375,376,377,378], Figure 25.

Ca_4-x_RE_6+x_(SiO_4_)_6-y_(PO_4_)_y_(O,F)_2_ can be actinide-host phases in HLW glass, glass-ceramic waste-forms, ceramic waste-forms and cements. The actinides can readily substitute in apatite for rare-earth elements as in Ca_2_(Nd,Cm,Pu)_8_(SiO_4_)_6_O_2_, and fission products are also readily incorporated. However, the solubility for tetravalent Pu may be limited without other charge compensating substitutions. 

Apatite has been proposed as a potential host phase for Pu and high-level actinide wastes. Structure: Hexagonal, Sp. gr. P6_3_/m or monoclinic, Sp. gr. P2_1_/b; may include: Na, K, Cs, Mg, Ca, Sr, Ba, Mn, Ni, Cd, Hg, Pb, Cr, Y, REE, Th, U, Si, P, V, As, S, F, Cl, OH and CO_3_. Ceramics were prepared by cold pressing and sintering, ρ = 95%; by HIP.

**Figure 26:**
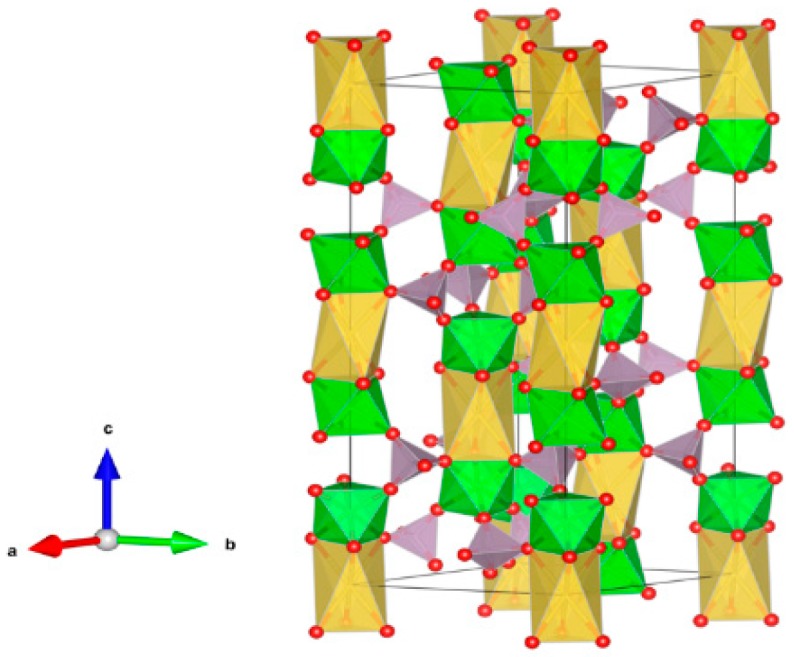
Sodium zirconium phosphate (NZP), NaZr_2_(PO_4_). Structure rhombohedral, Sp. gr. Rc, R3c, R3. Cations can be Li, Na, K, Rb, Cs, Cu and Ag; Mg, Ca, Mn, Zn, Sr and Ba; Mn, Co, Ni, Cu, Zn and Cd; Sc, Fe, Bi, Ce–Lu, Am and Cm; Zr, Hf, Th, U, Np and Pu; V, Nb, Sb and Ta; Ti, Ge, Zr, Hf, U, Np, Pu, Mo and Sn; Al, Sc, Cr, Fe, Ga, Y and In; Gd, Tb, Dy, Er and Yb; Mg; Na and K; Si, P, S, Mo and W.

26. Sodium zirconium phosphate (NZP) [17,18,19,20,21,22,23,24,87,89,155,209,211,293,379,380,381,382,383,384,385,386,387,388,389,390,391,392,393,394,395,396,397,398,399,400,401,402,403,404,405,406,407,408,409,410,411,412,413,414,415,416], Figure 26.

The first studies of materials with such a structure were carried out by the authors [379,380,381,382,383] in 1976–1987. They substantiated the crystal-chemical approach when choosing the composition of substances and their structural modifications with ion-transforming properties (Li+, Na+, etc.): NASICON, Langbeinite. Such materials have a frame structure: Na_1_ + _x_Zr_2_Si_x_P_3-x_O_12_, Na_3_M_2_ (PO_4_)_3_ (M = Sc, Cr, Fe), Na_5_Zr(PO_4_)_3_, Li_x_Fe_2_(WO_4_)_3_, Li_x_Fe_2_(MoO_4_)_3_. Elements in oxidation states 3–6 were introduced into the frame positions: Sc, Cr, Fe, Si, Zr, P, W and Mo. It was also the first time in 1987 that the rationale for the use of such structural analogs for the consolidation of HLW and transmutation of minoractinides [384] was presented. The development of such materials—Structural analogues of NASICON, NZP, Langbeinite—and their research, was continued in subsequent years.

NaZr_2_(PO_4_)_3_. The NZP structure can incorporate a complex variety of cations, including plutonium; a three dimensional network of corner-sharing ZrO_6_ octahedra and PO_4_ tetrahedra in which plutonium can substitute for Zr, as in Na(Zr,Pu)_2_(PO_4_)_3_. Complete substitution of Pu^4+^ for Zr has been demonstrated in NZP. Cs and Sr can substitute for Na, while fission products and actinides substitute for Zr in octahedral positions. P is tetrahedral. Phosphates with the mineral kosnarite structure (NaZr_2_(PO_4_)_3_ type, NZP) form a wide family. They can contain various cations in the oxidation state from 1+ to 5+. The structure consists of several positions and so many various cations can occupy it. These are MI = Li, Na, K, Rb, Cs; H, Cu(I) and Ag; MII = Mg, Ca, Sr, Ba, Mn, Co, Ni, Cu, Zn, Cd and Hg; MIII = Al, Ga, In, Sc, Y, La, Ce-Lu, Am, Cm, V, Cr, Fe, Sb and Bi; MIV = Ge, Sn, Ti, Zr, Hf, Mo, Ce, Th, U, Np and Pu; MV = Sb, Nb and Ta. Structure: Rhombohedral, Sp. gr. $R_{\bar{3}}c$, R3c, R3. This fact is extremely important, and can be useful for the synthesis of single-phase crystalline products of the solidification of radioactive waste whose cationic composition, as a rule, is extremely complicated. Ceramics were prepared by cold pressing and sintering (ρ = 80–98%), hot pressing (ρ = 96%) and Spark Plasma Sintering with high relative density (up to 98–99.9%).

**Figure 27:**
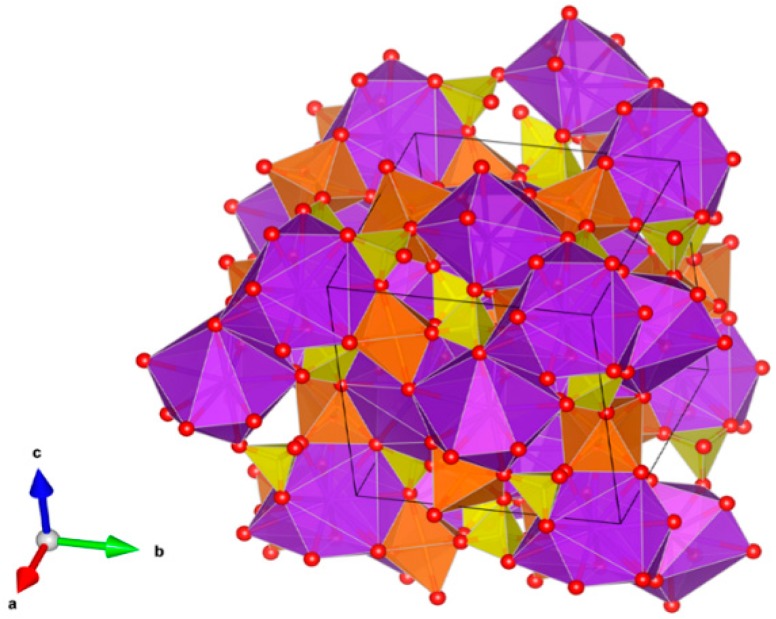
Langbeinite. K_2_Mg_2_(SO_4_)_3_. Structure cubic, Sp. gr. P2_1_3. Cations can be Na, K, Rb, Cs, Tl, NH_4_, Mg, Sr, Ba, Pb, Mn, Co, Ni, Zn, Al, Fe Cr, Ti^3+^, Ga, V^3+^, Rh, In, REE, Bi, Sn, Ti, Zr, Hf, P, Nb, Ta and S.

27. Langbeinite [18,87,89,211,293,416,417,418,419,420], Figure 27.

Langbeinite is a potassium magnesium sulfate mineral with the formula: K_2_Mg_2_(SO_4_)_3_. It may include much of cesium and other large 1- and 2-valent elements. The structure is a framework type, also as for its kosnarite structure. Structure: Cubic, Sp. gr. P2_1_3; may include: Na, K, Rb, Cs, Tl, NH_4_, Mg, Sr, Ba, Pb, Mn, Co, Ni, Zn, Al, Fe Cr, Ti^3+^, Ga, V^3+^, Rh, In, REE, Bi, Sn, Ti, Zr, Hf, P, Nb, Ta and S. Ceramics were prepared by cold pressing and sintering, ρ = 88%.

**Figure 28:**
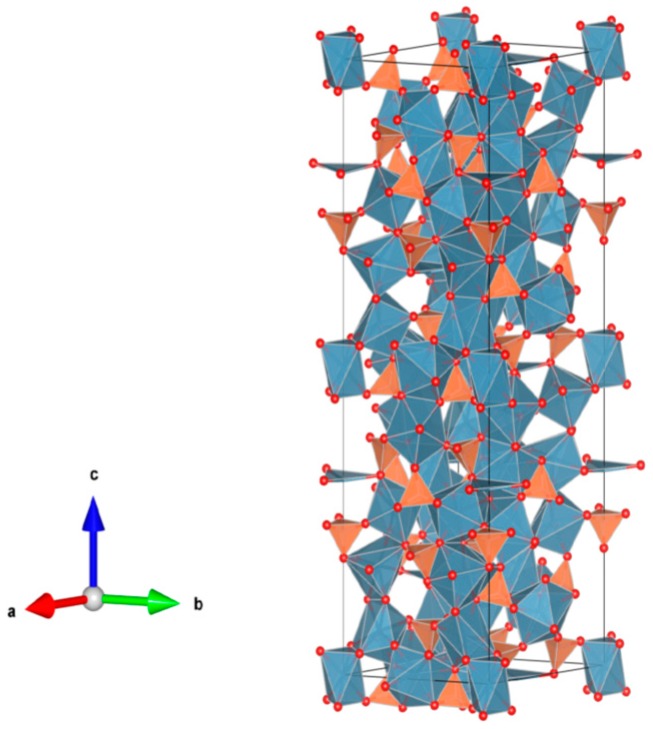
Whitlockite. Ca_3_(PO_4_)_2_. Structure trigonal, Sp. gr. R3c.Cations can be H, Li, Na, K, Cu, Mg, Ca, Sr, Ba, Al, Sc, Cr, Fe, Ga, In, La, Ce, Sm, Eu, Gd, Lu, Th, U and Pu.

28. Whitlockite [87,89,421,422,423,424,425,426,427,428,429,430,431,432], Figure 28.

Phosphates with the whitlockite structure (analog β-Ca_3_(PO_4_)_2_) were proposed as matrices for radioactive waste immobilization. Their origin is both biogenic and cosmogenic. Whitlockite samples from meteorites, rocks of the Moon, Mars and other cosmogenic bodies, preserve the crystalline form under the action of natural thermal “stress” and cosmic radiation. They contain small amounts of uranium and thorium, and it is presumed to contain plutonium. It is known to form isostructural compounds with H, Li, Na, K, Cu, Mg, Ca, Sr, Ba, Al, Sc, Cr, Fe, Ga, In, La, Ce, Sm, Eu, Gd, Lu, Th and Pu. Thermal stability is up to 1200 °C, thermal expansion up to 1 × 10^−5^ deg^−1^ (25–1000 °C) are close to Synroc and zirconolite; hydrothermal stable – leach rates at 90 °C up to 10^−5^ g·sm^−2^·day^−1^, radiation stable. Structure: Trigonal, Sp. gr. R3c. Ceramics were prepared by cold pressing and sintering (ρ = 92–97%) and Spark Plasma Sintering with high relative density (up to 95–98%).

**Figure 29:**
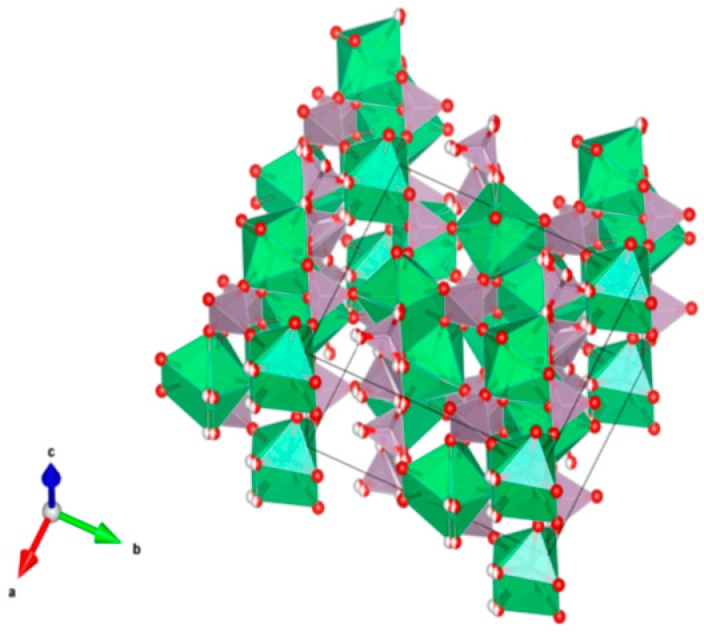
Thorium phosphate/Diphosphate (TPD). Th_4_(PO_4_)_4_P_2_O_7_. Structure orthorhombic. Sp. gr. Pbcm and Pcam. Cations can be U, Np, Pu, Am and Cm.

29. Thorium phosphate/Diphosphate (TPD) [155,244,336,337,433,434,435,436,437,438,439], Figure 29.

Th_4_(PO_4_)_4_P_2_O_7_; a unique compound for the immobilization of plutonium and uranium; partial substitution of Pu for Th has been demonstrated to up to 0.4 mole fraction, complete substitution is not possible. Structure: Orthorhombic, Sp. gr. Pbcm, Pcam, z = 2; may include: U, Np, Pu, Am and Cm. Ceramics were prepared by cold pressing and sintering (ρ = 87–93%).

### 4.5. Tungstate, Molybdates

**Figure 30:**
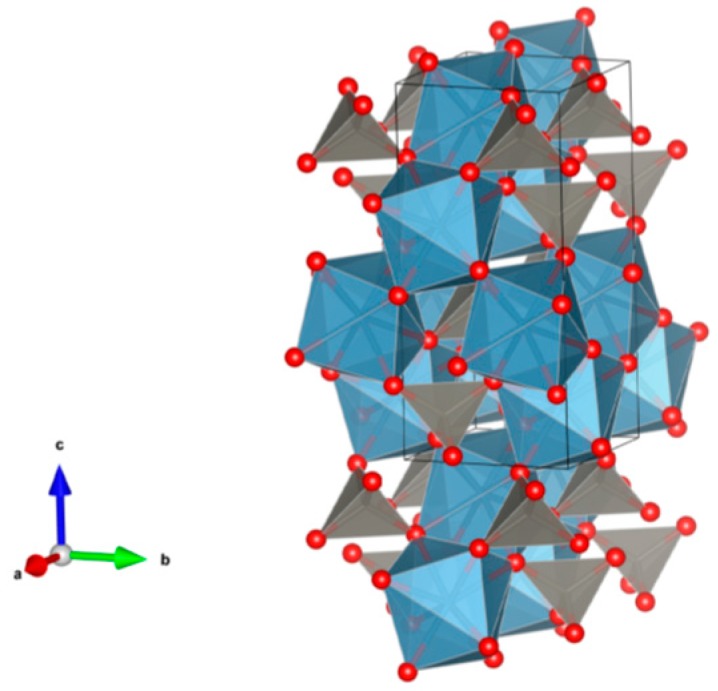
Scheelite. CaWO_4_. Structure tetragonal, Sp. gr. I4/c. Cations can be Li, Na, K, Rb, Cs, Tl, Ca, Sr, Ba, Mn, Cu, Fe, Ce, La–Lu, Y, Ge, Th, U, Np, Pu, Nb, Ta, V, Mo, W, Re and I.

30. Scheelite [89,440,441,442,443,444,445,446,447,448,449,450,451,452,453,454,455,456,457], Figure 30.

Materials with the structure of the scheelite mineral (calcium tungstate CaWO_4_) based on individual molybdates and tungstates and solid solutions may contain elements in oxidation degrees from 1+ to 7+: Li, Na, K, Rb, Cs and Tl; Ca, Sr, Ba, Mn and Cu; Fe, Ce, La–Lu and Y; Th, U, Np and Pu; Nb, Ta-in Ca-positions and Mo, W, Re, I, V and Ge in W-positions. The structural analog CaWO_4_ crystallizes in the tetragonal structure, Sp. gr. I4/c. The structure is constructed of CaO_8_ polyhedral and WO_4_ tetrahedrals connected through common oxygen vertices. For some compounds ceramics were prepared by the Spark Plasma Sintering (SPS) method, with a relative density of 92%.

## 5. Summary of Crystalline Ceramic Waste-forms

Crystalline materials including oxides-simple and complex, salts-silicates, phosphates, tungstates with various compositions and different structural modifications (30 structure forms) intended for nuclear waste immobilization were developed using various approaches and accounting for criteria of enough high durability (see e.g., [15,238,458,459,460]) requested for nuclear wasteforms. These are presented in Table 1.

Many of the compounds listed here have been studied and continue to be actively investigated by researchers led by the co-author of this work (Prof Orlova), including those with structures of garnet [185,189,190,191,192,193,194], P-pollucite [205,206,207,208,209,210,211,212,213,214,215], pollucite [214,215,293], monazite [141,352], sodium zirconium phosphate (NZP) [21,209,383,384,388,392,393,394,396,405,407,408,409,412,413,414,415,416,417,418,419], langbeinite [416,417,418,419], whitlockite [87,89,424,425,426,427,428,429,430] and scheelite [89,445,446]. Overall crystalline ceramics are characterized as much more durable compared with glasses of the same chemical composition e.g., the chemical durability of isomorph glasses is one to two orders of magnitude lower [458,459,460]. Nevertheless, the degree of the development of crystalline ceramics remains at the level of laboratory investigations rather than industrial use, except for SYNROC polyphase crystalline ceramic that is at the stage of the planned start of utilization by industry. Practically all structural forms developed (Table 1) are at the stage of obtaining compounds and their studies at the laboratory scale. The references [15,458,459,460] are also providing data on the acceptability of ionic size variability within the structure, and on chemical and radiation durability. 

From the analysis of the presented data of various compounds with various compositions and structural forms it is clear that researchers in the field of materials for nuclear waste immobilization have many variants available for work. While materials are mineral-like the principle ”from nature to nature” can be realized. Although many structures were included herewith, some could be missed, for example brannerite [15,99], which is currently considered for actinide immobilization [461]. Among most investigated structures one can note oxide ceramics. Some of crystalline ceramics such as monazite were synthesized using real (radioactive) actinides [15,235], whereas most of researchers use surrogate (non-radioactive) cations for investigations. 

## 6. Conclusions

1. Ceramic waste-forms for nuclear waste immobilization are investigated in different countries with a focus on improving environmental safety during storage, transport and disposal.
2. Inorganic compounds of oxide and salt character, having structural analogs with natural minerals, are being studied as most perspective materials for the immobilization of radioactive waste.
3. Approaches based on crystallochemistry principles are used when choosing the most favorable structural forms. They are based on the materials science concept “composition-structure-method of synthesis-property” accounting for the real task to be achieved. The basic principle is the isomorphism of cations and anions in compounds when choosing a real structure. Possible isomorphic substitutions in both cationic and anionic structural sites were considered in the works analyzed.
4. Crystalline ceramic waste-forms are intended to increase the environmental safety barrier when isolating radioactive materials (containing both actinides and fission products) from the biosphere. Among the methods of obtaining ceramic waste-forms, special attention in recent years is paid to sintering methods which ensure the formation of ceramics that, first, are almost non-porous e.g., have a relative density of up to 99.0–99.9% of theoretical, and, second, can be obtained within a small processing time e.g., within a few minutes (i.e., 2–3 min). These requirements are met by high-speed electric pulse sintering processes e.g., so-called Spark Plasma Sintering (SPS), although hot pressing enables the synthesis of very dense ceramics as well.

Professor Albina Orlova is working in the field of new inorganic materials used in nuclear chemistry for the rad-waste immobilization of dangerous isotopes, for actinide transmutation, as well for construction materials. She uses the structure properties and physico-chemical principles for the elaboration of new ceramics with mineral-like crystal forms. 

Professor Michael Ojovan is known for the connectivity-percolation theory of glass transition, the Sheffield model (two-exponential equation) of viscosity of glasses and melts, condensed Rydberg matter, metallic and glass-composite materials for nuclear waste immobilization, and self-sinking capsules to investigate Earth’s deep interior.

## Figures and Tables

**Table 1:** Crystalline ceramic materials as potential forms for nuclear waste immobilization.

Type of Chemical Compound		Structure		Compound Cations
		Structural Type	Syngony, Sp. gr.	
Oxide Compounds				
Simple oxides	SiO_2_	Silica	rhombohedral, R3	Li, Na, K. Mg, Ca, Mn, Cu, Ni, Pb B, Al, Fe, Cr, Ti, Zr, Te
	CeO_2_	Fluorite	cubic, Fm3m	Cs, Sr, Ce, Y, Zr, U, Th, Hf, Pu, U, Np
Complex oxides	A_2_B_2_O_7_	Pyrochlore	cubic, $Fd_{\bar{3}}m$	A: Na, Ca, U, Th, Y, Ln; B: Nb, Ta, Ti, Zr, Fe^3+^
	A_6_B_12_C_5_TX_40-x_	Murataite	cubic, $F4_{\bar{3}}m$	U, Np, Pu, Am, Cm, REE
	CaZrTi_2_O_7_	Zirconolite	trigonal C2/c	Gd, Hf, Ce, Th, U, Pu, Nb
	CaTiO_3_	Perovskite	cubic, Pm3m; rhombohedral, Pnma	Ca, Y, REE, Ti, Zr, U, Pu
	Ba_1.2_(Al,Ti)_8_O_16_	Hollandite	tetragonal, I4/m	Na, K, Cs, Mg, Ca, Ba, Al, Fe, Mn^3+^, Si, Ti, Mn^4+^
	^[8]^A_3_^[6]^B_2_[TiO_4_]_3_ .^[8]^(Ca,Gd, actinides)^[6]^Fe_2_^[4]^Fe_3_O_12_	Garnet	cubic, Ia3d	A, B: REE, An, Y, Mg, Ca, Fe^2+^, Mn^2+^; X: Cr^3+^, Fe^3+^, Al^3+^, Ga^3+^, Si^4+^, Ge^4+^, V^5+^
	(Sr,Pb,La,Ce,Y)(Ti,Fe^3+^,Mn,Mg,Zn,Cr,Al,Zr,Hf,U,V,Nb,Sn,Cu,Ni)_21_O_38_	Crichtonite	rhombohedral, R3	
	Na_2_Al_2_(Ti,Fe)_6_O_16_	Freudenbergitespinel based phase	monoclinic, C12/m1	Mg, Co, Ni, Zn, Al, Ti^3+^, Cr, Fe, Ga, Si, Nb
		P-Pollucite	cubic, I4_1_32	Li, Na, K, Rb, Cs, Tl, Be, Mg, Sr, Ba, Cd, Mn, Co, Ni, Cu, Zn, B, Al, Fe, Si, Ti, P, V, Nb, Ta
	ZrSiO_4_/ThSiO_4_/USiO_4_	Zircon/Thorite/Coffinite	tetragonal, I41/amd	REE, Th, U, Pu; Na, Mg, Ca, Mn, Co, Fe, Ti, P, V, Se, Mo
	CaTiSiO_5_ [CaTiO(SiO_4_)]	Titanite (sphene)	monoclinic, P_2_I/a	Mg, Ca, Sr, Ba, Mn, Al, Fe, Cr, Ce, Y, Zr, Th, F
	(REE,Ca)_5_(SiO_4_,PO_4_)_3_(OH,F)	Britholite (oxy-apatite)	monoclinic, sp. gr. P2_1_, hexagonal, P6_3_/m	Cs, Sr, B, Th, U, Np, Nd^3+^, La^3+^, Pu^3+^
Salt compounds				
Framework Silicates	(X_x/n_[(AlO_2_)_x_(SiO_2_)_y_]	Zeolites		Na, K, NH_4_^+^, Cs, Mg, Ca, Sr, Co, Fe, Ga, REE, Ti
	(Ca,Na)_2_Al_2_Si_4_O_12_·2H_2_O	Pollucite	cubic, Ia3d	Li, Na, K, Rb, Cs, Tl, Be, Mg, Sr, Ba, Cd, Mn, Co, Ni, Cu, Zn, B, Al, Fe, Si, Ti, P, V, Nb
	NaAlSiO_4_	Nepheline/Leucite	Nepheline: hexagonal, P6_3_; Leucite: tetragonal, I4_1_/a, I4_1_/acd; cubic, Ia3d	Li, Na, K, Rb, Cs, Be, Mg, Ca, Ba, Pb, Mn, Co, Ni, Al, Fe, Cr, Si, Ti, V
	Na_8_Cl_2_Al_6_Si_6_O_24_	Sodalite	cubic, $P_{\bar{4}}3n$	Na, K, Mg, Ca, Mn, Fe, Al, Si, Ti, Cl, SO_4_, CO_3_
	(Na,Ca,K)_6_[Al_6_Si_6_O_24_]((Na,Ca,K)_2_CO_3_)_1.6_·2.1H_2_O	Cancrinite	hexagonal, P6_3_	Cl^−^, SO_4_^2−^,
	[(Ca,Na,K,Ba)AlSiO_4_	Crystalline SilicoTitanate (CST)	cubic, sp. gr. Pm3m up to 105 °C, after tetragon. symm., sp. gr. I4/mcm or P4_2_/mcm	Na, K, Cs, Ca, Sr, Ba, Pb, Al, REE, Bi, Ti, Zr, Nb, Ta
	LiAl_3_Si_3_O_11_, NaAl_3_Si_3_O_11_, KAl_3_Si_3_O_11_, RbAl_3_Si_3_O_11_, CsAl_3_Si_3_O_11_, TlAl_3_Si_3_O_11_, Ca_0.5_□_0.5_Al_3_Si_3_O_11_, Sr_0.5_□_0.5_Al_3_Si_3_O_11_, Ba_0.5_□_0.5_Al_3_Si_3_O_11_, La_0.33_□_0.66_Al_3_Si_3_O_11_	Micas (Dehydroxylated)	monoclinic, C2/c	Cs, Rb, Ba, Mg, Fe^2+^, Fe^3+^, Mn, Li, Cr, Ti, V
Phosphates	CePO_4_	Monazite	monoclinic, P2_1_/n	Ce: Li, Na, K, Rb, Mg, Ca, Sr, Ba, Cd, Pb, Bi, Y, La, Pr, Nd, Sm, Eu, Gd, Tb, Yb, Am, Cm, Cf, Es, Ge, Zr, Th, Np, U, Pu; P: Cr, Si, Se, V, As, S
	YPO_4_	Xenotime	tetragonal, I4_1_/amd	Be, Ca, Al, Sc, La, Ce, Er, Dy–Lu, Zr, Th, U
	Ca_4-x_RE_6+x_(SiO_4_)_6-y_(PO_4_)_y_(O,F)_2_	Apatite	hexagonal, P6_3_/m; monoclinic, P2_1_/b	Na, K, Cs, Mg, Ca, Sr, Ba, Mn, Ni, Cd, Hg, Pb, Cr, Y, REE, Th, U, Si, P, V, As, S, F, Cl, OH, CO_3_
	NaZr_2_(PO_4_)_3_	Sodium zirconium phosphate (NZP)	rhombohedral, $R_{\bar{3}}c,$ R3c, R3	Li, Na, K, Rb, Cs; H, Cu(I), Ag, Mg, Ca, Sr, Ba, Mn, Co, Ni, Cu, Zn, Cd, Hg, Al, Ga, In, Sc, Y, La, Ce-Lu, Am, Cm, V, Cr, Fe, Sb, Bi, Ge, Sn, Ti, Zr, Hf, Mo, Ce, Th, U, Np, Pu, Sb, Nb, Ta
	K_2_Mg_2_(SO_4_)_3_	Langbeinite	cubic, P2_1_3	Na, K, Rb, Cs, Tl, NH4, Mg, Sr, Ba, Pb, Mn, Co, Ni, Zn, Al, Fe Cr, Ti^3+^, Ga, V^3+^, Rh, In, REE, Bi, Sn, Ti, Zr, Hf, P, Nb, Ta, S
	β-Ca_3_(PO_4_)_2_	Whitlockite	trigonal, R3c	H, Li, Na, K, Cu, Mg, Ca, Sr, Ba, Al, Sc, Cr, Fe, Ga, In, La, Ce, Sm, Eu, Gd, Lu, Th, Pu
	Th_4_(PO_4_)_4_P_2_O_7_	Thorium phosphate/Diphosphate (TPD)	orthorhombic, Pbcm, Pcam	U, Np, Pu, Am, Cm
Tungstates	CaWO_4_	Scheelite	tetragonal, I4/c	Ca: Li, Na, K, Rb, Cs, Tl; Ca, Sr, Ba, Mn, Cu; Fe, Ce, La-Lu, Y; Th, U, Np, Pu; Nb, Ta; W: Mo, Re, I, V, Ge
Aluminates	X(Al,Fe)_12_O_19_	Magnetoplumbite	hexagonal, P6_3_/mmc	Na, Cs, Mg, Sr, Ba, Pb, Mn, Co, Cu, Al, Fe, Sc, Y, La, Ce, Sm, Gd, Yb, Lu, An, Si, Ti, Sn
